# Food insecurity and food bank use: who is most at risk of severe food insecurity and who uses food banks?

**DOI:** 10.1017/S1368980024001393

**Published:** 2024-09-26

**Authors:** Elisabeth A Garratt, Beth Armstrong

**Affiliations:** 1 Sheffield Methods Institute, The University of Sheffield, Sheffield, UK; 2 Department of Geography, The University of Sheffield, Sheffield, UK

**Keywords:** Food insecurity, Poverty, Multivariate analyses, Emergency food, Health inequalities, Food bank, Inequality, UK, England, Wales and Northern Ireland

## Abstract

**Objectives:**

To identify (1) who experiences food insecurity of differing severity and (2) who uses food banks in England, Wales and Northern Ireland; (3) whether the same groups experience food insecurity and use food banks; and (4) to explore country- and region-level differences in food insecurity and food bank use.

**Design:**

This pooled cross-sectional study analysed the characteristics of adults experiencing food insecurity of differing severity using generalised ordinal logistic regression models and the characteristics of adults using food banks using logistic regression models, using data from three waves of the Food and You 2 surveys, 2021–2023.

**Setting:**

England, Wales and Northern Ireland.

**Participants:**

18 557 adults.

**Results:**

20·8 % of respondents experienced food insecurity in the past 12 months, and 3·6 % had used a food bank. Food insecurity was associated with income, working status, respondent age, family type, ethnicity, country, long-term health conditions, food hypersensitivity, urban-rural status and area-level deprivation. Severe food insecurity was concentrated among respondents with long-term health conditions and food hypersensitivities. Food bank use was more prevalent among food insecure respondents and unemployed and low-income respondents. Neither outcome showed clear geographical variation. Certain groups experienced an elevated likelihood of food insecurity but did not report correspondingly greater food bank use.

**Conclusions:**

Food insecurity is unevenly distributed, and its nutrition and health-related consequences demonstrate that food insecurity will intensify health inequalities. The divergence between the scale of food insecurity and food bank use strengthens calls for adequate policy responses.

Household food insecurity refers to people compromising the quantity or quality of food, experiencing anxiety about food supplies lasting and acquiring food in socially unacceptable ways^(1)^. Achieving zero hunger worldwide by 2030 is a UN Sustainable Development Goal, yet over the past decade, food insecurity increased in the UK^(2)^ and mainland Europe^(3)^. Latest figures from 2022 to 2023 show that across England, Wales and Northern Ireland (EWNI), 25 % of respondents were food insecure, and 4 % had used food banks in the previous 12 months. The significant and wide-ranging consequences of food insecurity make it a health, social and policy emergency. Research in high-income countries has linked food insecurity with a range of nutrition- and health-related outcomes, including reduced dietary quality^(4,5)^, poor general health^(6)^, heart disease^(7)^, diabetes^(8)^ and mental health problems in adults^(6)^ and children^(9)^.

Over the past decade, the unprecedented growth in UK food banks has established food insecurity as a key health and policy issue^(10)^. People using food banks are disproportionately food insecure^(11–14)^, financially disadvantaged^(11–15)^, male^(13)^, younger^(11,13,15)^, less educated^(15)^, have disabilities^(11,12)^ and are in lone-parent families^(11,12)^. However, previous analyses have primarily used descriptive analyses that are unable to isolate the characteristics associated with food bank use, leaving an incomplete understanding of who uses this form of emergency support.

Regular monitoring has been introduced in recent years, first biennially from 2016 in the Food and You survey and then annually from 2019 in the Family Resources Survey. This monitoring has contributed to a more complete picture of food insecurity in the UK, which is likewise concentrated in socio-economically disadvantaged groups as measured by low income^(2,11,16,17)^, unemployment^(2,11)^ and low education^(2,6,17)^. Food insecurity is also more common in households containing children^(2,6)^, especially lone-parent households^(6,11,17)^, among younger people^(2,6,11,16,17)^, ethnic minorities^(2,6)^ students^(6)^ and people in poor health^(11)^ or with disabilities^(2)^.

Existing research has typically overlooked differences in the severity of food insecurity, which ranges from worrying about food running out to not eating for a full day because there is not enough money for food. The United States Department of Agriculture Adult Food Security Survey classifies food security as high, marginal, low and very low^(18)^, where very low (‘severe’) food security is likely to have the most notable impact on nutrition- and health-related outcomes. To our knowledge, food insecurity of differing severity has to date only been explored using multivariate analyses once before, with Loopstra and colleagues reporting that low income, disability and unemployment were particularly relevant to severe food insecurity in 2004 and 2016^(2)^. More recently, the impacts of Brexit, the Russo-Ukraine war, the legacy of Covid-19 and the ongoing cost-of-living crisis have resulted in food insecurity increasing from 15 % in spring 2021 to 25 % in winter 2022–2023^(19)^. The high and growing scale of food insecurity makes it essential to examine the severity of food insecurity and the current groups most affected in greater detail.

## Research objectives

This research article has four research objectives: (1) To extend existing understandings of food insecurity by examining which groups of people currently experience food insecurity *of differing severity* in EWNI and thereby identify areas of particular vulnerability; (2) to identify who uses food banks in a nationally representative sample of EWNI; (3) to determine in a nationally representative sample whether the groups who experience food insecurity also use food banks; and (4) to explore geographical variations in food insecurity and food bank use, recognising that despite a UK food strategy^(20)^, devolution has contributed to policy differences at country and local government levels where more granular responses may be needed.

## Methods

### Data and sample

Food and You 2 (F&Y2) is a biannual, nationally representative household survey, commissioned by the Food Standards Agency, which has a policy remit for EWNI. F&Y2 is a push-to-web survey, which explores consumers’ food-related knowledge, attitudes and behaviours^(21)^. Following devolution, Food Standards Scotland is responsible for policy in Scotland and administers the Food in Scotland Survey.

The Food and You 2 sample uses stratified random sampling from the postcode address file, oversampling households in Wales and Northern Ireland to improve precision estimates. The sample was stratified by region, local authority and multiple deprivation score. Up to two adults (aged 16 years +) per household were invited to take part in the survey, making the data clustered at the household level. Sampling across survey waves was undertaken without replacement. Data from F&Y2 waves 4–6 offered the most recent data available (fieldwork: October 2021–January 2022; April–July 2022; October 2022–January 2023, *n* 18 557)^(22)^. Earlier waves of F&Y2 took place during Covid-19 restrictions, which may not be comparable with later waves.

### Variables and data preparation

Food insecurity was measured using the United States Department of Agriculture’s ten-item Adult Food Security Survey, a validated scale relating to the past 12 months. Marginal food security refers to 1–2 indications of food-access problems, commonly worry about food; low refers to compromised quality, variety or desirability without clear changes to diet or food intake, while very low food security captures demonstrably disrupted eating patterns and reduced food intake^(23)^. Food bank use was measured through the question, ‘In the last 12 months, have you, or anyone else in your household, received a free parcel of food from a food bank or other emergency food provider?’ We use the term ‘food bank use’ despite the question relating to emergency food providers in general because food banks are the dominant provider of food parcels, defined as food that is distributed for people to take away, then prepare and eat elsewhere^(24)^. While both food insecurity and food bank use are worded in relation to the household, they are nonetheless answered by individual respondents.

Predictor variables are outlined in Table 1. When exploring the outcome of food bank use, due to sparse data, we reduced food security to a binary predictor variable using the United States Department of Agriculture classifications of food secure (high and marginal) and food insecure (low and very low). We excluded respondents without data on food security status (*n* 714, 3·9 %) or food bank use (*n* 358, 2·17 %) from relevant analyses. The sample size for food bank analyses is smaller because questions about food bank use were asked of online but not postal respondents in wave 4. To maximise the sample size and reduce bias, we used multiple imputations to reduce the quantity of missing data on the predictor variables (less than 7 % for each of the predictor variables, except income where missingness was higher at 24·8 %). Missing data were imputed by chained equations on the basis of the other predictor variables, food security status and survey mode^(25)^, under the missing at random assumption. All findings were replicated in sensitivity analyses undertaken without imputation (results available on request).

**Table 1:** Summary of variables

Variable	Measurement level	Block
Outcome variables		
Food security status	Household	
Food bank use	Household	
Predictor variables		
Gender	Individual	Block 1, demographics
Age group	Individual	
Ethnic group	Individual	
Household composition	Household	
Survey wave	Household	
Banded annual income	Household	Block 2, financial factors
Employment status	Individual	
Presence of a long-term health condition	Individual	Block 3, health
Presence of a food hypersensitivity	Individual	
Multiple deprivation quintile	Household	Block 4, local area characteristics
Country	Household	
Region	Household	
Urban-rural status	Household	

### Analytic approach

We explored the characteristics of respondents (a) experiencing food insecurity and (b) using food banks using bivariate and multivariate statistical models. Descriptive statistics with tests of association are available in supplementary materials. Reflecting the ordinal nature of the food security variable, we specified generalised ordinal logistic regression models (‘gologit models’) using the gologit2 command available within Stata 17 software. Gologit models are equivalent to simultaneously specifying a set of binary logistic regressions that combine categories of the response option, in this case, to explore the odds of high, marginal and low food security against very low food security^(26)^. The gologit2 command allows a blend of predictor variables that satisfy the parallel-lines (or proportional odds) assumption, where the same coefficient describes the relationship between predictor variables and food insecurity of differing severity, combined with variables that do not satisfy this assumption and where different-sized coefficients are needed to predict food insecurity of differing severity^(27)^.

We weighted all models to adjust for the sampling design. We adjusted for household-level clustering because (a) adults living in larger households have a lower probability of inclusion and (b) demographic and food-related characteristics are likely to be correlated within households. We controlled for the survey wave as descriptive statistics demonstrated that food insecurity varied over time. When exploring geographical variation in food insecurity and food bank use, we specified models that included interaction terms between country and demographic predictors. We did not consider interaction terms including region due to model complexity and the small number of respondents in each region. We entered predictor variables into models in four blocks (see Table 1). The final models are partial proportional odds models in which most – but not all – coefficients are the same across the outcomes of high, marginal, low or very low food security.

## Results

### Who experiences food security of differing severity?

Descriptive statistics demonstrated that 20·8 % of respondents experienced food insecurity in the past 12 months (11·0 % low, 9·8 % very low). Food insecurity rose from 17·6 % (8·8 million adults) in October 2021–January 2022 (10·3 % low, 7·3 % very low) to 24·6 % (12·4 million adults) in October 2022–January 2023 (12·2 % low, 12·4 % very low).

Table 2 shows bivariate associations between food security status and demographic characteristics, while Table 3 shows multivariate analyses identifying characteristics associated with food insecurity after mutual adjustment for other predictors. Results from blocks 1, 2 and 3 are available in supplementary materials. Model results are presented as OR, where OR and CI above one identify characteristics that are associated with a greater likelihood of lower levels of food security than the reference category. Conversely, OR and CI below one identify characteristics that are associated with a reduced likelihood of lower levels of food security than the reference category.

**Table 2:** Bivariate ordinal logistic regression analyses predicting food security status of differing severity, showing odds ratios and 95 % confidence intervals, *n* 17 843

	High *v*. marginal, low and very low food security		High and marginal *v*. low and very low food security		High, marginal and low *v*. very low food security	
	OR	95 % CI	OR	95 % CI	OR	95 % CI
Survey wave						
Wave 4 (Oct 2021–Jan 2022)	1·00	1·00, 1·00	1·00··	1·00, 1·00	1·00	1·00, 1·00
Wave 5 (April–July 2022)	1·20*	1·04, 1·38	1·20*	1·04, 1·38	1·20*	1·04, 1·38
Wave 6 (Oct 2022–Jan 2023)	1·52***	1·32, 1·76	1·52***	1·32, 1·76	1·52***	1·32, 1·76
Gender						
Male	1·00	1·00, 1·00	1·00	1·00, 1·00	1·00	1·00, 1·00
Female	1·09	0·99, 1·20	1·09	0·99, 1·20	1·09	0·99, 1·20
Age group						
16–24	1·00	1·00, 1·00	1·00	1·00, 1·00	1·00	1·00, 1·00
25–34	0·76*	0·61, 0·95	0·76*	0·61, 0·95	0·76*	0·61, 0·95
35–44	0·64***	0·52, 0·80	0·64***	0·52, 0·80	0·64***	0·52, 0·80
45–54	0·44***	0·35, 0·55	0·44***	0·35, 0·55	0·44***	0·35, 0·55
55–64	0·27***	0·22, 0·34	0·27***	0·22, 0·34	0·27***	0·22, 0·34
65–74	0·19***	0·16, 0·24	0·19***	0·16, 0·24	0·19***	0·16, 0·24
75+	*0·16* ***	*0·12, 0·21*	*0·12* ***	*0·08, 0·17*	*0·06* ***	*0·03, 0·11*
Household composition						
One adult no children	1·00	1·00, 1·00	1·00	1·00, 1·00	1·00	1·00, 1·00
Couple no children	0·70***	0·60, 0·80	0·70***	0·60, 0·80	0·70***	0·60, 0·80
Couple with children	1·53***	1·30, 1·80	1·53***	1·30, 1·80	1·53***	1·30, 1·80
Lone parent	4·90***	3·68, 6·51	4·90***	3·68, 6·51	4·90***	3·68, 6·51
Other no children	0·98	0·82, 1·19	0·98	0·82, 1·19	0·98	0·82, 1·19
Other with children	1·92***	1·51, 2·45	1·92***	1·51, 2·45	1·92***	1·51, 2·45
Ethnicity†						
White	1·00	1·00, 1·00	1·00	1·00, 1·00	1·00	1·00, 1·00
Mixed	2·78***	1·98, 3·90	2·78***	1·98, 3·90	2·78***	1·98, 3·90
Asian or Asian British	*2·12* ***	*1·67, 2·68*	*1·91* ***	*1·47, 2·48*	*1·07*	*0·72, 1·59*
Black or black British	1·99***	1·44, 2·75	1·99***	1·44, 2·75	1·99***	1·44, 2·75
Other ethnic group	1·83*	1·04, 3·19	1·83*	1·04, 3·19	1·83*	1·04, 3·19
Income‡						
Less than £19 000	1·00	1·00, 1·00	1·00	1·00, 1·00	1·00	1·00, 1·00
£19 000–£31 999	*0·49* ***	*0·42, 0·56*	*0·42* ***	*0·36, 0·50*	*0·43* ***	*0·35, 0·53*
£32 000–£63 999	*0·27* ***	*0·24, 0·31*	*0·20* ***	*0·17, 0·24*	*0·17* ***	*0·13, 0·23*
£64 000 and above	*0·10* ***	*0·08, 0·12*	*0·06* ***	*0·04, 0·08*	*0·02* ***	*0·01, 0·04*
Employment status						
Working	1·00	1·00, 1·00	1·00	1·00, 1·00	1·00	1·00, 1·00
Student	1·87***	1·46, 2·40	1·87***	1·46, 2·40	1·87***	1·46, 2·40
Retired	*0·36* ***	*0·32, 0·41*	*0·27* ***	*0·23, 0·33*	*0·20* ***	*0·15, 0·26*
Unemployed	5·34***	3·82, 7·48	5·34***	3·82, 7·48	5·34***	3·82, 7·48
Unable to work due to poor health	5·08***	3·91, 6·60	5·08***	3·91, 6·60	5·08***	3·91, 6·60
Homemaker	2·02***	1·60, 2·53	2·02***	1·60, 2·53	2·02***	1·60, 2·53
Other	1·56**	1·16, 2·10	1·56**	1·16, 2·10	1·56**	1·16, 2·10
Long-term health condition status						
No long-term health condition	1·00	1·00, 1·00	1·00	1·00, 1·00	1·00	1·00, 1·00
Has a long-term health condition	*1·62* ***	*1·45, 1·80*	*2·03* ***	*1·79, 2·31*	*2·75* ***	*2·31, 3·29*
Food hypersensitivity status§						
No food hypersensitivity	1·00	1·00, 1·00	1·00	1·00, 1·00	1·00	1·00, 1·00
Has a food hypersensitivity	*1·21* **	*1·05, 1·38*	*1·22* *	*1·04, 1·43*	*1·54* ***	*1·25, 1·91*
Urban/rural classification						
Urban	1·00	1·00, 1·00	1·00	1·00, 1·00	1·00	1·00, 1·00
Rural	0·55***	0·49, 0·63	0·55***	0·49, 0·63	0·55***	0·49, 0·63
Country						
England	1·00	1·00, 1·00	1·00	1·00, 1·00	1·00	1·00, 1·00
Wales	1·19**	1·07, 1·33	1·19**	1·07, 1·33	1·19**	1·07, 1·33
Northern Ireland	1·10	1·00, 1·22	1·10	1·00, 1·22	1·10	1·00, 1·22
Region						
North-East England	1·00	1·00, 1·00	1·00	1·00, 1·00	1·00	1·00, 1·00
North-West England	0·98	0·73, 1·32	0·98	0·73, 1·32	0·98	0·73, 1·32
Yorkshire and the Humber	1·14	0·83, 1·57	1·14	0·83, 1·57	1·14	0·83, 1·57
West Midlands	1·15	0·84, 1·56	1·15	0·84, 1·56	1·15	0·84, 1·56
East Midlands	1·00	0·73, 1·37	1·00	0·73, 1·37	1·00	0·73, 1·37
East of England	0·73*	0·53, 0·99	0·73*	0·53, 0·99	0·73*	0·53, 0·99
South-East England	0·76	0·57, 1·03	0·76	0·57, 1·03	0·76	0·57, 1·03
South-West England	0·72*	0·52, 0·99	0·72*	0·52, 0·99	0·72*	0·52, 0·99
Greater London	1·01	0·74, 1·36	1·01	0·74, 1·36	1·01	0·74, 1·36
Wales	1·10	0·84, 1·44	1·10	0·84, 1·44	1·10	0·84, 1·44
Northern Ireland	1·01	0·78, 1·32	1·01	0·78, 1·32	1·01	0·78, 1·32
Multiple deprivation						
Quintile 1 – most deprived	1·00	1·00, 1·00	1·00	1·00, 1·00	1·00	1·00, 1·00
Quintile 2	0·52***	0·44, 0·62	0·52***	0·44, 0·62	0·52***	0·44, 0·62
Quintile 3	0·46***··	0·39, 0·56	0·46***··	0·39, 0·56	0·46***··	0·39, 0·56
Quintile 4	0·33***	0·28, 0·39	0·33***	0·28, 0·39	0·33***	0·28, 0·39
Quintile 5 – least deprived	0·23***	0·20, 0·28	0·23***	0·20, 0·28	0·23***	0·20, 0·28

* *P* < 0·05.

** *P* < 0·01.

*** *P* < 0·001.

† Ethnicity defined using the Office for National Statistics harmonised categories.

‡ Income is annual non-equivalised household income.

§ Hypersensitivity captures the presence or absence of a self-reported food allergy, intolerance and/or coeliac disease.

The English Index of Multiple Deprivation (2019), Welsh Index of Multiple Deprivation (2019) and Northern Ireland Multiple Deprivation Measure (2017) are not directly comparable between countries as they are constructed from different indicators in England, Wales and Northern Ireland. To make the analyses more comparable between respondents from different countries, we included the country as a control variable in analyses containing deprivation quintile.

Coefficients that do not satisfy the proportional odds assumption and that have therefore been allowed to vary across different levels of food security are denoted by italics.

**Table 3:** Multivariate ordinal logistic regression analyses predicting food security status of differing severity (block 4), showing odds ratios and 95 % confidence intervals, *n* 17 843

	High *v*. marginal, low and very low food security		High and marginal *v*. low and very low food security			High, marginal and low *v*. very low food security	
	OR	95 % CI	OR		95 % CI	OR	95 % CI
Survey wave							
Wave 4 (Oct 2021–Jan 2022)	1·00	1·00, 1·00	1·00		1·00, 1·00	1·00	1·00, 1·00
Wave 5 (April–July 2022)	1·32***	1·13, 1·53	1·32***		1·13, 1·53	1·32***	1·13, 1·53
Wave 6 (Oct 2022–Jan 2023)	*1·75* ***	*1·50, 2·04*	*1·74* ***		*1·46, 2·08*	*1·83* ***	*1·46, 2·29*
Gender							
Male	1·00	1·00, 1·00	1·00		1·00, 1·00	1·00	1·00, 1·00
Female	0·93	0·82, 1·04	0·93		0·82, 1·04	0·93	0·82, 1·04
Age group							
16–24	1·00	1·00, 1·00	1·00		1·00, 1·00	1·00	1·00, 1·00
25–34	0·79	0·58, 1·08	0·79		0·58, 1·08	0·79	0·58, 1·08
35–44	0·64**	0·48, 0·87	0·64**		0·48, 0·87	0·64**	0·48, 0·87
45–54	0·45***	0·33, 0·62	0·45***		0·33, 0·62	0·45***	0·33, 0·62
55–64	0·25***	0·19, 0·35	0·25***		0·19, 0·35	0·25***	0·19, 0·35
65–74	0·23***	0·17, 0·32	0·23***		0·17, 0·32	0·23***	0·17, 0·32
75 +	*0·20* ***	*0·14, 0·30*	*0·15* ***		*0·09, 0·24*	*0·08* ***	*0·04, 0·17*
Household composition							
One adult no children	1·00	1·00, 1·00	1·00		1·00, 1·00	1·00	1·00, 1·00
Couple no children	0·95	0·81, 1·12	0·95		0·81, 1·12	0·95	0·81, 1·12
Couple with children	1·51***	1·23, 1·85	1·51***		1·23, 1·85	1·51***	1·23, 1·85
Lone parent	2·54***	1·84, 3·52	2·54***		1·84, 3·52	2·54***	1·84, 3·52
Other no children	1·03	0·83, 1·28	1·03		0·83, 1·28	1·03	0·83, 1·28
Other with children	1·78***	1·34, 2·36	1·78***		1·34, 2·36	1·78***	1·34, 2·36
Ethnicity							
White	1·00	1·00, 1·00	1·00		1·00, 1·00	1·00	1·00, 1·00
Mixed	1·73*	1·13, 2·65	1·73*		1·13, 2·65	1·73*	1·13, 2·65
Asian or Asian British	*1·07*	*0·80, 1·45*	*1·03*		*0·75, 1·41*	*0·58* **	*0·38, 0·87*
Black or black British	1·22	0·76, 1·96	1·22		0·76, 1·96	1·22	0·76, 1·96
Other ethnic group	0·91	0·46, 1·80	0·91		0·46, 1·80	0·91	0·46, 1·80
Annual household income (non-equivalised)							
Less than £19 000	1·00	1·00, 1·00	1·00		1·00, 1·00	1·00	1·00, 1·00
£19 000–£31 999	*0·41* ***	*0·35, 0·48*	*0·37* ***		*0·31, 0·45*	*0·43* ***	*0·34, 0·54*
£32 000–£63 999	*0·17* ***	*0·14, 0·21*	*0·15* ***		*0·12, 0·18*	*0·15* ***	*0·11, 0·20*
£64 000 and above	*0·06* ***	*0·04, 0·07*	*0·04* ***		*0·03, 0·06*	*0·02* ***	*0·01, 0·04*
Employment status							
Working	1·00	1·00, 1·00	1·00		1·00, 1·00	1·00	1·00, 1·00
Student	0·44***	0·31, 0·64	0·44***		0·31, 0·64	0·44***	0·31, 0·64
Retired	0·37***	0·31, 0·46	0·37***		0·31, 0·46	0·37***	0·31, 0·46
Unemployed	1·50*	1·03, 2·20	1·50*		1·03, 2·20	1·50*	1·03, 2·20
Unable to work due to poor health	1·29	0·95, 1·76	1·29		0·95, 1·76	1·29	0·95, 1·76
Homemaker	1·03	0·82, 1·29	1·03		0·82, 1·29	1·03	0·82, 1·29
Other	0·98	0·71, 1·35	0·98		0·71, 1·35	0·98	0·71, 1·35
Long-term health condition status							
No long-term health condition	1·00	1·00, 1·00	1·00		1·00, 1·00	1·00	1·00, 1·00
Has a long-term health condition	*1·69* ***	*1·48, 1·93*	*2·06* ***		*1·76, 2·41*	*2·44* ***	*1·98, 3·01*
Food hypersensitivity status							
No food hypersensitivity	1·00	1·00, 1·00	1·00		1·00, 1·00	1·00	1·00, 1·00
Has a food hypersensitivity	*1·21* *	*1·03, 1·42*	*1·20*		*1·00, 1·43*	*1·53* ***	*1·20, 1·95*
Urban/rural classification							
Urban	1·00	1·00, 1·00	1·00		1·00, 1·00	1·00	1·00, 1·00
Rural	0·77***	0·66, 0·89	0·77***		0·66, 0·89	0·77***	0·66, 0·89
Country							
England	1·00	1·00, 1·00	1·00		1·00, 1·00	1·00	1·00, 1·00
Wales	1·25***	1·11, 1·41	1·25***		1·11, 1·41	1·25***	1·11, 1·41
Northern Ireland	1·04	0·93, 1·17	1·04		0·93, 1·17	1·04	0·93, 1·17
Multiple deprivation							
Quintile 1 – most deprived	1·00	1·00, 1·00		1·00	1·00, 1·00	1·00	1·00, 1·00
Quintile 2	0·67***	0·56, 0·80		0·67***	0·56, 0·80	0·67***	0·56, 0·80
Quintile 3	0·82*	0·67, 1·00		0·82*	0·67, 1·00	0·82*	0·67, 1·00
Quintile 4	0·68***	0·56, 0·83		0·68***	0·56, 0·83	0·68***	0·56, 0·83
Quintile 5 – least deprived	0·61***	0·50, 0·75		0·61***	0·50, 0·75	0·61***	0·50, 0·75

* *P* < 0·05.

** *P* < 0·01.

*** *P* < 0·001.

Table 3 shows that in multivariate analyses, compared with wave 4, food insecurity was more prevalent in wave 5 (OR: 1·32; 95 % CI: 1·13, 1·53), while the higher odds of food insecurity in wave 6 were magnified for severe food security (OR: 1·83; 95 % CI: 1·46, 2·29) compared with high to marginal food security (OR: 1·75; 95 % CI: 1·50, 2·04).

Compared with 16–24-year-old respondents, the odds of experiencing higher levels of food insecurity became progressively lower from age 35. Among those over 75, the odds of experiencing food insecurity decreased with greater severity (OR for high to marginal food security: 0·20, 95 % CI: 0·14, 0·30; OR for severe food security: 0·08, 95 % CI: 0·04, 0·17). All household types with children experienced a higher prevalence of food insecurity than one-person households; this pattern was especially stark for lone parents (OR: 2·54; 95 % CI: 1·84, 3·52). Compared with White respondents, mixed-race respondents were more likely to experience food insecurity (OR: 1·73; 95 % CI: 1·13, 2·65), while Asian respondents were significantly less likely to experience very low food insecurity (OR: 0·58; 95 % CI: 0·38, 0·87).

Unsurprisingly, higher incomes were associated with lower odds of more severe food insecurity: compared with respondents with household incomes of less than £19 000 per year, those with annual household incomes of £64 000 and over had odds of 0·06 (95 % CI: 0·04, 0·07) for experiencing low food security and 0·02 (95 % CI: 0·01, 0·04) for experiencing very low food security. Compared with working respondents, food insecurity was less prevalent among students (OR: 0·44; 95 % CI: 0·31, 0·64) and retired respondents (OR: 0·37; 95 % CI: 0·31, 0·46) and more prevalent among unemployed respondents (OR: 1·50; 95 % CI: 1·03, 2·20). Having a long-term health condition (LTHC) was associated with higher odds of experiencing food insecurity compared with respondents without an LTHC, and the odds were greater for low (OR: 2·06; 95 % CI: 1·76, 2·41) and very low (OR: 2·44; 95 % CI: 1·98, 3·01) food security. The same pattern was evident for food hypersensitivities (OR for high to marginal food security: 1·21, 95 % CI: 1·03, 1·42; OR for low to very low food security: 1·53, 95 % CI: 1·20, 1·95) compared with respondents without a food hypersensitivity. Higher levels of food insecurity were more prevalent among respondents in Wales (OR: 1·25; 95 % CI: 1·11, 1·41) than in England and less prevalent for those living in rural (OR: 0·77; 95 % CI: 0·66, 0·89) than urban areas and less deprived areas (OR for quintile 5: 0·61; 95 % CI: 0·50, 0·75). Food insecurity of differing severity was not associated with gender in bivariate or multivariate analyses.

### Who uses food banks?

In descriptive statistics, 3·6 % of respondents used a food bank in the previous 12 months – equating to approximately 1·8 million adults – rising to 17·9 % among those experiencing food insecurity. Food bank use fluctuated non-significantly over the survey waves.

Table 4 shows associations between food bank use and demographic characteristics, beginning with bivariate associations and then entering further predictors in thematic blocks, using multivariate analyses to identify characteristics associated with food bank use after mutual adjustment for other predictors. As before, OR with CI above one identify characteristics that are associated with a greater likelihood of food bank use compared with the reference category, while OR and CI below one identify characteristics associated with a lower likelihood of food bank use.

**Table 4:** Logistic regression models predicting food bank use showing odds ratios and 95 % confidence intervals, *n* 11 161

	Bivariate associations		Block 1: Demographics		Block 2: Demographics plus financial characteristics			Block 3: Demographics plus financial characteristics plus health		Block 4: Demographics plus financial characteristics plus health plus local characteristics	
	OR	95 % CI	OR	95 % CI	OR	95 % CI		OR	95 % CI	OR	95 % CI
Intercept	Variable, from 0·01 to 0·12		0·05***	0·02, 0·12	0·08***	0·03, 0·20		0·07***	0·03, 0·18	0·10***	0·03, 0·28
Survey wave											
Wave 4 (Oct 2021–Jan 2022)	1·00	.,.	1·00	.,.	1·00	.,.		1·00	.,.	1·00	.,.
Wave 5 (April–July 2022)	0·72	0·46, 1·12	0·65	0·41, 1·05	0·68	0·42, 1·10		0·68	0·42, 1·12	0·70	0·43, 1·13
Wave 6 (Oct 2022–Jan 2023)	0·85	0·53, 1·38	0·63	0·39, 1·04	0·67	0·40, 1·13		0·69	0·40, 1·17	0·72	0·42, 1·21
Food security status											
High or marginal food security	1·00	.,.	1·00	.,.	1·00	.,.		1·00	.,.	1·00	.,.
Low or very low food security	13·57***	8·53, 21·60	11·22***	6·95, 18·11	4·93***	3·04, 7·99		4·52***	2·82, 7·26	4·41***	2·75, 7·06
Gender											
Male	1·00	.,.	1·00	.,.	1·00	.,.		1·00	.,.	1·00	.,.
Female	1·08	0·76, 1·54	1·03	0·71, 1·48	0·91	0·61, 1·36		0·89	0·59, 1·34	0·90	0·60, 1·34
Age group											
16–24	1·00	.,.	1·00	.,.	1·00	.,.		1·00	.,.	1·00	.,.
25–34	0·42**	0·24, 0·76	0·40**	0·22, 0·75	0·39*	0·19, 0·81		0·39*	0·19, 0·81	0·36**	0·17, 0·75
35–44	0·51*	0·30, 0·89	0·55*	0·31, 0·99	0·60	0·30, 1·18		0·59	0·29, 1·18	0·56	0·28, 1·13
45–54	0·33***	0·19, 0·59	0·49*	0·27, 0·90	0·49	0·23, 1·02		0·49	0·23, 1·03	0·50	0·24, 1·05
55–64	0·22***	0·11, 0·42	0·40**	0·20, 0·80	0·33**	0·15, 0·73		0·32**	0·15, 0·71	0·35**	0·16, 0·77
65–74	0·10***	0·04, 0·23	0·26**	0·10, 0·63	0·39	0·10, 1·45		0·37	0·10, 1·42	0·37	0·10, 1·40
75 +	0·20*	0·05, 0·75	0·48	0·11, 2·06	0·87	0·16, 4·60		0·78	0·14, 4·29	0·86	0·15, 4·77
Household composition											
One adult no children	1·00	.,.	1·00	.,.	1·00	.,.		1·00	.,.	1·00	.,.
Couple no children	0·54*	0·32, 0·89	0·56*	0·33, 0·97	0·87	0·50, 1·51		0·89	0·51, 1·54	0·88	0·51, 1·53
Couple with children	1·09	0·65, 1·86	0·64	0·34, 1·19	1·15	0·60, 2·21		1·23	0·64, 2·37	1·33	0·69, 2·59
Lone parent	4·20***	2·26, 7·80	1·62	0·77, 3·40	1·98	0·90, 4·35		2·03	0·91, 4·51	2·20	0·99, 4·87
Other no children	0·84	0·43, 1·67	0·52	0·26, 1·04	0·87	0·42, 1·77		0·86	0·42, 1·76	0·89	0·45, 1·79
Other with children	1·09	0·51, 2·30	0·45*	0·20, 0·99	0·75	0·34, 1·68		0·75	0·33, 1·69	0·78	0·34, 1·76
Ethnicity											
White	1·00	.,.	1·00	.,.	1·00	.,.		1·00	.,.	1·00	.,.
Ethnic minority	2·39**	1·41, 4·05	1·62	0·93, 2·84	1·44	0·83, 2·51		1·53	0·87, 2·68	1·45	0·79, 2·64
Household income											
Less than £19 000	1·00	.,.			1·00	.,.		1·00	.,.	1·00	.,.
£19 000–£31 999	0·22***	0·13, 0·37			0·43**	0·24, 0·77		0·45**	0·25, 0·82	0·22***	0·13, 0·37
£32 000 and above	0·05***	0·03, 0·12			0·16***	0·06, 0·41		0·18***	0·07, 0·46	0·05***	0·03, 0·12
Employment status											
Working	1·00	.,.			1·00	.,.		1·00	.,.	1·00	.,.
Student	3·89***	1·90, 7·96			1·12	0·43, 2·95		1·09	0·40, 2·92	1·15	0·43, 3·08
Retired	0·47	0·21, 1·05			0·55	0·18, 1·65		0·52	0·17, 1·60	0·56	0·18, 1·76
Unemployed	12·86***	7·38, 22·38			3·84***	2·10, 7·04		3·48***	1·87, 6·48	3·76***	2·01, 7·03
Unable to work due to poor health	13·77***	8·54, 22·22			4·45***	2·54, 7·77		3·48***	1·82, 6·68	3·58***	1·85, 6·95
Homemaker	4·48***	2·59, 7·74			2·03	0·99, 4·15		1·86	0·89, 3·87	1·93	0·92, 4·07
Other	4·47***	2·28, 8·80			2·83**	1·39, 5·77		2·75**	1·35, 5·57	2·68**	1·27, 5·68
Long-term health condition status											
No long-term health condition	1·00	.,.						1·00	.,.	1·00	.,.
Has a long-term health condition	2·92***	1·98, 4·31						1·51	0·92, 2·50	1·45	0·87, 2·41
Food hypersensitivity status											
No food hypersensitivity	1·00	.,.						1·00	.,.	1·00	.,.
Has a food hypersensitivity	1·18	0·74, 1·88						1·03	0·58, 1·82	1·06	0·60, 1·85
Urban/rural classification											
Urban	1·00	.,.								1·00	.,.
Rural	0·55*	0·32, 0·95								1·21	0·69, 2·13
Country											
England	1·00	.,.								1·00	.,.
Wales	1·06	0·74, 1·50								0·94	0·63, 1·41
Northern Ireland	1·27	0·92, 1·76								1·31	0·87, 1·96
Index of multiple deprivation											
Quintile 1 (most deprived)	1·00	.,.								1·00	.,.
Quintile 2	0·37***	0·24, 0·58								0·47**	0·28, 0·79
Quintile 4	0·34***	0·19, 0·60								0·53*	0·28, 1·00
Quintile 4	0·22***	0·10, 0·48								0·54	0·24, 1·21
Quintile 5 (least deprived)	0·11***	0·05, 0·24								0·37*	0·17, 0·83

* *P* < 0·05.

** *P* < 0·01.

*** *P* < 0·001.

In the final model (block 4), the strongest predictor of food bank use was food insecurity, where respondents experiencing food insecurity were over four times as likely to use food banks compared with those not experiencing food insecurity (OR: 4·41; 95 % CI: 2·75, 7·06). Respondents who were unemployed (OR: 3·76; 95 % CI: 2·01, 7·03) or unable to work due to ill health (OR: 3·58, 95 % CI: 1·85, 6·95) were over three times as likely to use food banks compared with working respondents, while respondents with ‘other’ working status also had elevated odds of food bank use (OR: 2·68; 95 % CI: 1·27, 5·68). Food bank use was less prevalent at higher incomes (OR at annual incomes exceeding £32 000: 0·05, 95 % CI: 0·03, 0·12) compared with respondents with incomes of less than £19 000 per year and in the least deprived compared with the most deprived local areas (OR at quintile 5: 0·37; 95 % CI: 0·17, 0·83).

The higher odds of food bank use among lone parents, ethnic minority respondents, students, homemakers and respondents with LTHC and the lower odds of food bank use in retired people, couples without children and respondents in rural areas in bivariate analysis attenuated to non-significance in multivariate analyses. These findings demonstrate the importance of accounting for compositional differences. Age also became inconsistently related to food bank use in multivariate analyses. Food bank use was not significantly associated with survey wave, gender, food hypersensitivity and country in bivariate or multivariate analyses.

These findings were directly replicated in sensitivity analyses predicting food bank use from severe food insecurity (see online supplementary material, Supplemental Table S8). They were additionally broadly replicated in sensitivity analyses predicting food bank use without controlling for food security status (see online supplementary material, Supplemental Table S9). Notable are two exceptions: without controlling for food security, food bank use was more prevalent in lone parents than one-person households (OR: 2·66; 95 % CI: 1·23, 5·75) and in respondents with LTHC compared with those without (OR: 1·92; 95 % CI: 1·16, 3·18).

### Geographical variations in food insecurity and food bank use

Our analyses revealed no clear differences in the predictors of food insecurity and food bank use across EWNI (see online supplementary material, Supplemental Tables S6 and S7).

## Discussion

### Who experiences food security of differing severity?

Our first objective was to capitalise on the availability of contemporary, nationally representative data on food insecurity in EWNI to examine the demographic and geographical characteristics of people experiencing food insecurity of differing severity in EWNI. Our multivariate analyses revealed that food insecurity is concentrated in economically disadvantaged groups. Unsurprisingly, higher levels of food insecurity were more prevalent in lower-income households^(2,11,16)^, and higher incomes were particularly protective against severe food insecurity. In line with previous research, food insecurity was more prevalent among unemployed respondents^(2,11)^, who are likely to be exposed to an often unreliable benefit system^(28)^. Replicating existing research, food insecurity was more prevalent in households containing children^(2,6)^, especially lone-parent households^(2,6,11,17)^. As these latter associations held after controlling for banded income, we suggest that non-financial factors may be important; for example, time pressures that can translate into higher food costs may be especially acute in lone-parent households.

Higher levels of food insecurity was more prevalent among younger people^(2,14,29)^, where factors including precarious work and reduced benefit entitlements are known to create vulnerabilities^(30)^. Mixed-race respondents were more likely to experience food insecurity of differing severity, while Asian respondents had a lower prevalence of very low food security, which could reflect familial and community support^(31)^, and demonstrates the value of exploring food insecurity of differing severity. Worryingly, the concentration of food insecurity – particularly its more severe forms – among respondents with LTHC and food hypersensitivities corroborates evidence of vulnerability among those with disabilities^(32)^ and may reflect higher dietary costs for those with hypersensitivities^(33)^. Compared with employed respondents, higher levels of food insecurity were less prevalent among retired people, consistent with Canadian research that identified a drop in food insecurity at pension age^(34)^. Students were also less likely to experience food insecurity. This finding contrasts with recent evidence^(35)^ and demonstrates the importance of multivariate analyses that controls for income and other characteristics. Students may be protected by specific support or lower living costs elsewhere.

The lower prevalence of food insecurity in less deprived local areas suggests a protective role for the immediate local environment – even after controlling for income – while the lower prevalence of food insecurity in rural areas contradicts European evidence^(36)^. Contributing to mixed evidence on the topic^(2,6)^, gender was not associated with food insecurity.

### Who uses food banks?

Our second objective was to identify who uses food banks in a nationally representative sample of EWNI. Overall, 3·6 % of respondents used a food bank in the previous 12 months. The strongest predictor of food bank use was, unsurprisingly, food security status, with people experiencing food insecurity being over four times more likely to use food banks. This finding replicates existing bivariate evidence^(37)^ of severe food insecurity among people using food banks and reinforces evidence that food bank use is driven by need and not – as critics have suggested^(38)^ – by supply-side factors or opportunism.

Predictably, food bank use was more prevalent among lower-income groups^(12)^. After controlling for income, food bank use was concentrated in those who were unemployed^(12)^ or unable to work due to ill health^(13)^. Independent of their resources, these groups may have more ready access to referral agencies or be better able to make use of emergency food provision with restricted opening hours^(39)^. As noted above, not working exposes people to an often unreliable benefit system, where food bank use is linked to receipt of state benefits^(11,14)^ and benefit sanctions^(40)^. A broad but inconsistent association between younger age and food bank use replicates previous evidence^(11,13,15)^, while the absence of associations by gender contradicts evidence for higher food bank use in men^(13)^. Our findings are important in light of past evidence linking food bank use with these characteristics using less rigorous analyses that did not mutually adjust for other characteristics. Taken together, our findings provide evidence that food bank use is most closely associated with financial disadvantage and experiences of food insecurity.

### Do the same groups experience food insecurity and use food banks?

Noting that the prevalence of food insecurity far outstrips food bank use, our third objective was to determine in a nationally representative sample whether the same groups experience food insecurity and use food banks. Looking first at similarities, food insecurity was the strongest predictor of food bank use. Both food insecurity and food bank use were more prevalent at lower incomes and among unemployed and younger people. After accounting for food insecurity status, we did not find an independent association between food bank use and being a lone parent or living in a rural or deprived area. Our findings suggest that food bank use is predicted primarily by the concentration of food insecurity in these groups but that other unmeasured factors are also likely to play a role. Having an LTHC was associated with a greater prevalence of food insecurity – particularly its more severe forms – however, having an LTHC was not associated with food bank use after employment status was controlled. Based on these findings, we suggest that higher food bank use among people with LTHC is driven by a greater prevalence of food insecurity and lower workforce participation in this group^(32)^.

We also identified some key differences in the prevalence of food insecurity and food bank use for different groups. Food insecurity was more prevalent in waves 5 and 6, in households containing children, in respondents with food hypersensitivity and in Wales, without an accompanying rise in food bank use. These patterns may be understood through reference to a recently proposed conceptual framework in which the likelihood of food insecurity leading to food bank use is impacted by a combination of people’s thoughts and feelings about food bank use and the operational and landscape features of community support available^(24)^. For example, food bank provision may be unsuited to people with hypersensitivities^(41)^, while more broadly, the cost-of-living crisis has left some food banks struggling to source adequate supplies to meet growing demand^(42)^, which could explain the rise in food insecurity over time without a parallel rise in food bank use.

Conversely, some groups had a lower prevalence of food insecurity but did not report lower levels of food bank use. Despite rural and less deprived areas having a lower prevalence of food insecurity, they did not have comparatively lower levels of food bank use, which contrasts with past evidence for the highly uneven provision of food banks in rural areas^(43)^. Retired people and students were similarly less likely to be food insecure but no less likely to use food banks, which may again reflect good access to referral agencies and availability of food banks among these groups. Finally, some characteristics were associated with food bank use only. Respondents unable to work due to ill health did not experience a higher prevalence of food insecurity but were more likely to use food banks, which may reflect support-seeking behaviours that connect these respondents with referral agencies or greater potential to access food banks during opening hours. Discrepancies between food insecurity and food bank use by different ethnic groups potentially reflect the methodological consequences of aggregating diverse groups during analyses and highlight the importance of detailed research by ethnicity^(44)^.

### Geographical variations in food insecurity and food bank use

Our fourth objective was to explore country- and region-level variation in food insecurity and food bank use. Respondents in Wales were more likely to experience food insecurity, but food bank use did not vary by country. As above, this pattern could reflect unmet needs in the form of limited access to referral agencies or food banks or the availability of other forms of community food provision that people may access instead of food banks. Region was not associated with either food insecurity or food bank use. The absence of interactions by country demonstrates that food insecurity and food bank use are associated with the same demographic characteristics across countries.

### Policy implications

The latest data show that one in five adults in EWNI are food insecure, unquestionably identifying this phenomenon as a critical and intensifying nutrition and health issue. A robust policy response to UK food insecurity is long overdue, yet the current approach of allowing the third sector to take responsibility is demonstrably inadequate, as evidenced by uneven food bank use^(13,45)^, which in any case does not alleviate food insecurity^(46)^. Our findings contribute to these discussions by providing further robust evidence that certain groups – households containing children, respondents with food hypersensitivities and in Wales – experience an elevated likelihood of food insecurity but did not report correspondingly greater food bank use. Thus, while the commitment of emergency food providers is laudable, they should not be relied upon as central responses to food insecurity.

Indeed, our results reinforce existing evidence for the protective role of higher incomes, especially in relation to severe food insecurity. This finding is especially relevant in the current financial climate, where shocks such as the cost-of-living crisis are disproportionately disruptive to low-income households. In the UK National Food Strategy, Henry Dimbleby recommended that ‘ideally, of course, the true cost of eating healthily should be calculated into benefits payments’^(20)^. Yet, the final strategy contains no measures to directly protect incomes from either work or benefits, instead endorsing peripheral measures such as community healthy eating programmes and a limited extension of free school meals that excludes some households experiencing low or very low food security. The strategy has consequently been criticised for failing to meaningfully address food poverty^(47)^. We urge ministers to align wages and benefits to meet the costs of a healthy diet, noting that food insecurity is not associated with cooking confidence or food management strategies^(29)^, so individually focused interventions are unlikely to be effective. Instead, structural interventions to enhance incomes are needed. Additional support is needed for people with LTHC and lone parents, groups who are disproportionately likely to experience food insecurity and whose potential to work is commonly constrained. Further targeted support is also needed for people with LTHC and food hypersensitivities, who are most at risk of severe food insecurity.

### Limitations

This study is inevitably affected by certain limitations posed by the F&Y2 dataset. As a private household survey, the current analyses will have underestimated the overall prevalence and depth of food insecurity and the scale of food bank use, which is concentrated among vulnerably housed and roofless people^(48)^ who are unlikely to have been surveyed in F&Y2. Our analyses are nonetheless valuable in demonstrating the recent growth of food insecurity and in identifying the current characteristics of housed people experiencing food insecurity and food bank use. By asking respondents about their receipt of food parcels, the F&Y2 data offer an incomplete picture of alternative low- and no-cost food provision, such as social supermarkets and community cafes, and informal strategies such as bin diving. Further data are needed to contextualise the current evidence on food bank use within the wider alternative food landscape. We do however caution against allowing a more inclusive understanding of alternative food provision to detract from the overarching goal to build financial resilience to allow people to source food in mainstream, socially acceptable ways.

### Future research directions

It would be fruitful to explore the persistence of food insecurity in response to psychological, economic, social and relational factors. Such analyses would be especially valuable in light of the high prevalence of food insecurity among lone parents and respondents with LTHC. Panel models using longitudinal data such as the UK’s Understanding Society would enable a closer approximation to identifying causality and enable an exploration of the frequency of food bank use, where approximately half of the recipients access food banks once^(14,49)^, but repeat visits are unevenly distributed across different groups^(49)^. While F&Y2 asks about the frequency of food bank use, its sample size does not enable detailed analyses on the topic. Sophisticated multivariate analyses of repeat food bank use have the potential to distil groups of short- and longer-term food bank recipients who may benefit from differentiated policy interventions.

By using ordinal logistic regression models, our analyses offer insights not only into the demographic characteristics associated with food insecurity but also the relevance of these characteristics to the *severity* of food insecurity. Noting the elevated odds of severe food insecurity among respondents with LTHC and food hypersensitivities, future research could valuably explore whether there exist specific demographic profiles of people experiencing food insecurity of differing severities. Such insights could inform bespoke policy interventions.

### Conclusions

Our descriptive analyses found that 20·8 % of respondents experienced food insecurity in the past 12 months and 3·6 % had used a food bank over the past 12 months, rising to 17·9 % among those experiencing food insecurity. Our multivariate statistical analyses revealed that food insecurity in EWNI was concentrated among economically disadvantaged groups (those with low incomes and unemployed respondents), younger respondents, households containing children (especially lone-parent households), mixed-race respondents, in Wales and respondents with LTHC or food hypersensitivities. Worryingly, having an LTHC or food hypersensitivity was associated with more severe experiences of food insecurity, a consideration that has received very little research attention to date and that demonstrates enhanced vulnerability among people with existing poor health, where good nutrition may be particularly important.

Food bank use was similarly more prevalent among respondents experiencing food insecurity and unemployed and low-income respondents. However, we observed some differences, where certain respondents – households containing children, with food hypersensitivities and in Wales – experienced an elevated likelihood of food insecurity but did not report an elevated likelihood of food bank use. These discrepancies suggest a divergence between need and crisis support that merits further research attention. No clear geographical variation was evident across outcomes.

Worryingly, the rise in food insecurity, especially severe food insecurity, was not accompanied by an increase in food bank use over the survey period, demonstrating a divergence between need and crisis support. To reduce food insecurity and its negative nutrition and health consequences, policy measures are urgently needed to meaningfully strengthen people’s financial resources and provide enhanced support to vulnerable groups.

## Supporting information

Garratt and Armstrong supplementary material 1 Garratt and Armstrong supplementary material

Garratt and Armstrong supplementary material 2 Garratt and Armstrong supplementary material

## Supplementary material

For supplementary material accompanying this paper, visit https://doi.org/10.1017/S1368980024001393.
