# Food insecurity and food bank use: who is most at risk of severe food insecurity and who uses food banks? – CORRIGENDUM

**DOI:** 10.1017/S136898002500028X

**Published:** 2025-04-02

**Authors:** Elisabeth A Garratt, Beth Armstrong

The Authors regret the omission of funding information for this article, which has now been added.
