# Supplementary material for: Food insecurity and food bank use: who is most at risk of severe food insecurity and who uses food banks?
Source: Public Health Nutr. 2024 Sep 26;27(1):e174. doi: 10.1017/S1368980024001393 (PMC11505125; doi:10.1017/S1368980024001393)
Supplement: Garratt and Armstrong supplementary material 1 — Garratt and Armstrong supplementary material [file S1368980024001393sup001.docx]

# **Supplementary materials**

Supplementary table S1: Weighted bivariate associations between food security status and predictor variables, pooling waves 4 to 6 (n=16,659)

|  | **High food security** | **Marginal food security** | **Low food security** | **Very low food security** | **p value from chi-squared test of association** |
| --- | --- | --- | --- | --- | --- |
|  | **n=11,016** | **n=2,184** | **n=1,826** | **n=1,632** |  |
| Survey wave | | | | | |
| Wave 4 (Oct 2021 - Jan 2022) | 70.4% | 12.0% | 10.3% | 7.3% | **<0.001** |
| Wave 5 (April - July 2022) | 66.6% | 13.3% | 10.4% | 9.7% |  |
| Wave 6 (Oct 2022 - Jan 2023) | 61.4% | 14.0% | 12.2% | 12.4% |  |
| Gender | | | | | |
| Male | 67.5% | 12.5% | 10.4% | 9.7% | **<0.001** |
| Female | 65.4% | 13.6% | 11.3% | 9.7% |  |
| Age group | | | | | |
| 16-24 | 46.2% | 18.3% | 17.3% | 18.3% | **<0.001** |
| 25-34 | 54.0% | 16.0% | 14.9% | 15.1% |  |
| 35-44 | 57.6% | 16.0% | 14.7% | 11.7% |  |
| 45-54 | 67.6% | 12.2% | 9.9% | 10.3% |  |
| 55-64 | 76.6% | 9.3% | 7.8% | 6.3% |  |
| 65-74 | 81.8% | 10.0% | 5.4% | 2.7% |  |
| 75 + | 84.9% | 9.1% | 4.8% | 1.3% |  |
| Household composition | | | | | |
| One adult no children | 68.5% | 13.0% | 8.4% | 10.1% | **<0.001** |
| Couple no children | 74.6% | 10.9% | 8.5% | 6.1% |  |
| Couple with children | 56.6% | 16.2% | 14.4% | 12.8% |  |
| Lone parent | 29.9% | 16.4% | 19.2% | 34.5% |  |
| Other no children | 67.8% | 13.0% | 10.5% | 8.7% |  |
| Other with children | 52.6% | 15.5% | 16.5% | 15.4% |  |
| Ethnicity | | | | | |
| White | 69.3% | 12.2% | 9.0% | 9.5% | **<0.001** |
| Mixed | 43.7% | 15.5% | 24.3% | 16.6% |  |
| Asian or Asian British | 51.2% | 17.7% | 21.0% | 10.2% |  |
| Black or Black British | 48.0% | 22.5% | 19.9% | 9.5% |  |
| Other ethnic group | 53.8% | 15.8% | 17.3% | 13.1% |  |
| Annual household income (non equivalised) | | | | | |
| Less than £19,000 | 42.9% | 13.9% | 19.8% | 23.4% | **<0.001** |
| £19,000 - £31,999 | 59.6% | 15.6% | 12.6% | 12.3% |  |
| £32,000 - £63,999 | 73.4% | 13.3% | 8.4% | 4.8% |  |
| £64,000 and above | 89.8% | 6.5% | 3.0% | 0.6% |  |
| Employment status | | | | | |
| Working | 65.8% | 13.7% | 11.5% | 9.0% | **<0.001** |
| Student | 49.4% | 19.2% | 15.9% | 15.5% |  |
| Retired | 84.7% | 9.0% | 4.4% | 1.9% |  |
| Unemployed | 30.5% | 11.2% | 20.3% | 38.0% |  |
| Unable to work due to poor health | 29.4% | 14.3% | 20.0% | 36.3% |  |
| Homemaker | 50.4% | 14.4% | 16.9% | 18.3% |  |
| Other | 56.6% | 14.4% | 14.1% | 15.0% |  |
| Long-term health condition status | | | | | |
| No long-term health condition | 70.3% | 13.4% | 10.1% | 6.2% | **<0.001** |
| Has a long-term health condition | 59.3% | 11.5% | 12.5% | 16.8% |  |
| Food hypersensitivity status | | | | | |
| No food hypersensitivity | 67.4% | 12.8% | 11.0% | 8.8% | **<0.001** |
| Has a food hypersensitivity | 63.3% | 14.1% | 9.5% | 13.2% |  |
| Urban/rural classification | | | | | |
| Urban | 63.6% | 13.9% | 12.0% | 10.6% | **<0.001** |
| Rural | 76.1% | 10.1% | 7.0% | 6.8% |  |
| Country | | | | | |
| England | 66.4% | 13.1% | 10.9% | 9.6% | **<0.001** |
| Wales | 62.9% | 13.6% | 11.2% | 12.4% |  |
| Northern Ireland | 63.9% | 14.2% | 11.8% | 10.1% |  |
| Region | | | | | |
| North-East England | 64.6% | 13.5% | 11.2% | 10.7% | **<0.001** |
| North-West England | 65.1% | 13.3% | 12.3% | 9.3% |  |
| Yorkshire and the Humber | 62.6% | 12.4% | 11.0% | 14.0% |  |
| West Midlands | 61.2% | 15.7% | 11.2% | 12.0% |  |
| East Midlands | 64.5% | 13.7% | 11.2% | 10.6% |  |
| East of England | 71.4% | 11.4% | 9.5% | 7.7% |  |
| South-East England | 70.4% | 11.7% | 9.2% | 8.7% |  |
| South-West England | 71.8% | 11.8% | 7.8% | 8.6% |  |
| Greater London | 63.6% | 14.4% | 14.2% | 7.8% |  |
| Wales | 62.9% | 13.6% | 11.2% | 12.4% |  |
| Northern Ireland | 63.9% | 14.2% | 11.8% | 10.1% |  |
| Multiple deprivation | | | | | |
| Quintile 1 - Most deprived | 47.4% | 17.4% | 17.8% | 17.4% | **<0.0 01** |
| Quintile 2 | 63.5% | 14.3% | 11.5% | 10.7% |  |
| Quintile 3 | 66.8% | 11.7% | 11.5% | 10.0% |  |
| Quintile 4 | 73.1% | 12.0% | 8.0% | 6.9% |  |
| Quintile 5 - Least deprived | 79.3% | 10.2% | 6.3% | 4.2% |  |

Significant associations as determined by the chi-squared test of association are denoted in bold text

Supplementary table S2: Weighted bivariate associations between emergency food receipt in the past 12 months and predictor variables, pooling waves 4 to 6 (n=10,913)

|  | **Received emergency food (%)** | **Not received emergency food (%)** | **p value from chi-squared test of association** |
| --- | --- | --- | --- |
|  | **n=395** | **n=10,518** |  |
| Survey wave | | | |
| Wave 4 (Oct 2021-Jan 2022) | 4.2% | 95.8% | 0.636 |
| Wave 5 (April - July 2022) | 3.0% | 97.0% |  |
| Wave 6 (Oct 2022 - Jan 2023) | 3.7% | 96.3% |  |
| Food security status | | | |
| High | 0.7% | 99.3% | **<0.001** |
| Marginal | 3.7% | 96.4% |  |
| Low | 7.5% | 92.5% |  |
| Very low | 20.6% | 79.4% |  |
| Gender | | | |
| Male | 3.5% | 96.6% | 0.085 |
| Female | 3.6% | 96.4% |  |
| Age group | | | |
| 16-24 | 8.2% | 91.8% | **<0.001** |
| 25-34 | 3.6% | 96.4% |  |
| 35-44 | 4.7% | 95.3% |  |
| 45-54 | 3.9% | 96.1% |  |
| 55-64 | 2.2% | 97.8% |  |
| 65-74 | 1.4% | 98.7% |  |
| 75 + | 1.3% | 98.7% |  |
| Household composition | | | |
| One adult no children | 4.4% | 95.7% | **<0.001** |
| Couple no children | 2.2% | 97.8% |  |
| Couple with children | 4.8% | 95.2% |  |
| Lone parent | 14.7% | 85.3% |  |
| Other no children | 3.7% | 96.3% |  |
| Other with children | 4.5% | 95.5% |  |
| Ethnicity | | | |
| White | 2.9% | 97.1% | **<0.001** |
| Mixed | 9.8% | 90.2% |  |
| Asian or Asian British | 7.8% | 92.2% |  |
| Black or Black British | 11.0% | 89.0% |  |
| Other ethnic group | 3.2% | 96.8% |  |
| Annual household income (non equivalised) | | | |
| Less than £19,000 | 11.6% | 88.4% | **<0.001** |
| £19,000 - £31,999 | 2.7% | 97.3% |  |
| £32,000 and above | 0.3% | 99.7% |  |
| Employment status | | | |
| Working | 2.2% | 97.9% | **<0.001** |
| Student | 7.5% | 92.5% |  |
| Retired | 1.0% | 99.0% |  |
| Unemployed | 19.0% | 81.0% |  |
| Unable to work due to poor health | 20.6% | 79.5% |  |
| Homemaker | 9.4% | 90.6% |  |
| Other | 7.5% | 92.5% |  |
| Long-term health condition status | | | |
| No long-term health condition | 2.2% | 97.8% | **<0.001** |
| Has a long-term health condition | 6.4% | 93.6% |  |
| Food hypersensitivity status | | | |
| No food hypersensitivity | 3.5% | 96.5% | **0.015** |
| Has a food hypersensitivity | 4.0% | 96.0% |  |
| Urban/rural classification | | | |
| Urban | 4.0% | 96.0% | **0.001** |
| Rural | 2.3% | 97.7% |  |
| Country | | | |
| England | 3.6% | 96.4% | **0.044** |
| Wales | 3.8% | 96.2% |  |
| Northern Ireland | 4.3% | 95.7% |  |
| Region | | | |
| North-East England | 3.1% | 96.9% | **0.002** |
| North-West England | 3.9% | 96.1% |  |
| Yorkshire and the Humber | 4.6% | 95.4% |  |
| West Midlands | 3.9% | 96.1% |  |
| East Midlands | 4.5% | 95.5% |  |
| East of England | 3.2% | 96.8% |  |
| South-East England | 2.2% | 97.8% |  |
| South-West England | 2.9% | 97.1% |  |
| Greater London | 4.3% | 95.7% |  |
| Wales | 3.8% | 96.2% |  |
| Northern Ireland | 4.3% | 95.7% |  |
| Multiple deprivation | | | |
| Quintile 1 - Most deprived | 8.1% | 91.9% | **<0.001** |
| Quintile 2 | 3.8% | 96.2% |  |
| Quintile 3 | 2.8% | 97.2% |  |
| Quintile 4 | 2.5% | 97.5% |  |
| Quintile 5 - Least deprived | 1.1% | 98.9% |  |

Significant associations as determined by the chi-squared test of association are denoted in bold text

Supplementary table S3: Ordinal logistic regression analyses predicting food security status of differing severity, Block 1, showing odds ratios and standard errors, n=17,843

|  | **High vs marginal, low and very low food security** | **High and marginal vs low and very low food security** | **High, marginal and low vs very low food security** |
| --- | --- | --- | --- |
| Survey wave | | | |
| Wave 4 (Oct 2021-Jan 2022) | 1.00 [1.00,1.00] | 1.00 [1.00,1.00] | 1.00 [1.00,1.00] |
| Wave 5 (April - July 2022) | 1.20* [1.04,1.38] | 1.20* [1.04,1.38] | 1.20* [1.04,1.38] |
| Wave 6 (Oct 2022 - Jan 2023) | *1.51*** [1.31,1.75]* | *1.54*** [1.30,1.81]* | *1.61*** [1.30,2.00]* |
| Gender | | | |
| Male | 1.00 [1.00,1.00] | 1.00 [1.00,1.00] | 1.00 [1.00,1.00] |
| Female | 1.11 [1.00,1.23] | 1.11 [1.00,1.23] | 1.11 [1.00,1.23] |
| Age group | | | |
| 16-24 | 1.00 [1.00,1.00] | 1.00 [1.00,1.00] | 1.00 [1.00,1.00] |
| 25-34 | 0.77* [0.60,0.97] | 0.77* [0.60,0.97] | 0.77* [0.60,0.97] |
| 35-44 | 0.55*** [0.44,0.70] | 0.55*** [0.44,0.70] | 0.55*** [0.44,0.70] |
| 45-54 | 0.43*** [0.34,0.54] | 0.43*** [0.34,0.54] | 0.43*** [0.34,0.54] |
| 55-64 | 0.30*** [0.24,0.38] | 0.30*** [0.24,0.38] | 0.30*** [0.24,0.38] |
| 65-74 | 0.21*** [0.16,0.26] | 0.21*** [0.16,0.26] | 0.21*** [0.16,0.26] |
| 75 + | *0.17*** [0.12,0.23]* | *0.12*** [0.08,0.18]* | *0.06*** [0.03,0.11]* |
| Household composition | | | |
| One adult no children | 1.00 [1.00,1.00] | 1.00 [1.00,1.00] | 1.00 [1.00,1.00] |
| Couple no children | 0.57*** [0.48,0.66] | 0.57*** [0.48,0.66] | 0.57*** [0.48,0.66] |
| Couple with children | 0.78* [0.64,0.94] | 0.78* [0.64,0.94] | 0.78* [0.64,0.94] |
| Lone parent | 2.53*** [1.88,3.41] | 2.53*** [1.88,3.41] | 2.53*** [1.88,3.41] |
| Other no children | 0.56*** [0.46,0.69] | 0.56*** [0.46,0.69] | 0.56*** [0.46,0.69] |
| Other with children | 0.93 [0.71,1.22] | 0.93 [0.71,1.22] | 0.93 [0.71,1.22] |
| Ethnicity | | | |
| White | 1.00 [1.00,1.00] | 1.00 [1.00,1.00] | 1.00 [1.00,1.00] |
| Mixed | 1.97*** [1.39,2.79] | 1.97*** [1.39,2.79] | 1.97*** [1.39,2.79] |
| Asian or Asian British | *1.49** [1.15,1.92]* | *1.36* [1.02,1.80]* | *0.76 [0.51,1.14]* |
| Black or Black British | 1.31 [0.91,1.88] | 1.31 [0.91,1.88] | 1.31 [0.91,1.88] |
| Other ethnic group | 1.30 [0.64,2.65] | 1.30 [0.64,2.65] | 1.30 [0.64,2.65] |

* p < 0.05, ** p < 0.01, *** p < 0.001

Coefficients that do not satisfy the proportional odds assumption and that have therefore been allowed to vary across different levels of food security have been denoted by italics

Supplementary table S4: Ordinal logistic regression analyses predicting food security status of differing severity, Block 2 showing odds ratios and standard errors, n=17,843

|  | **High vs marginal, low and very low food security** | **High and marginal vs low and very low food security** | **High, marginal and low vs very low food security** |
| --- | --- | --- | --- |
| Survey wave | | | |
| Wave 4 (Oct 2021-Jan 2022) | 1.00 [1.00,1.00] | 1.00 [1.00,1.00] | 1.00 [1.00,1.00] |
| Wave 5 (April - July 2022) | 1.27** [1.10,1.48] | 1.27** [1.10,1.48] | 1.27** [1.10,1.48] |
| Wave 6 (Oct 2022 - Jan 2023) | *1.68*** [1.44,1.97]* | *1.67*** [1.40,1.99]* | *1.71*** [1.36,2.14]* |
| Gender | | | |
| Male | 1.00 [1.00,1.00] | 1.00 [1.00,1.00] | 1.00 [1.00,1.00] |
| Female | 0.96 [0.86,1.08] | 0.96 [0.86,1.08] | 0.96 [0.86,1.08] |
| Age group | | | |
| 16-24 | 1.00 [1.00,1.00] | 1.00 [1.00,1.00] | 1.00 [1.00,1.00] |
| 25-34 | 0.83 [0.61,1.13] | 0.83 [0.61,1.13] | 0.83 [0.61,1.13] |
| 35-44 | 0.67** [0.49,0.90] | 0.67** [0.49,0.90] | 0.67** [0.49,0.90] |
| 45-54 | 0.46*** [0.34,0.62] | 0.46*** [0.34,0.62] | 0.46*** [0.34,0.62] |
| 55-64 | 0.25*** [0.18,0.34] | 0.25*** [0.18,0.34] | 0.25*** [0.18,0.34] |
| 65-74 | 0.24*** [0.17,0.33] | 0.24*** [0.17,0.33] | 0.24*** [0.17,0.33] |
| 75 + | *0.21*** [0.15,0.32]* | *0.16*** [0.10,0.25]* | *0.09*** [0.04,0.18]* |
| Household composition | | | |
| One adult no children | 1.00 [1.00,1.00] | 1.00 [1.00,1.00] | 1.00 [1.00,1.00] |
| Couple no children | 0.91 [0.78,1.06] | 0.91 [0.78,1.06] | 0.91 [0.78,1.06] |
| Couple with children | 1.30** [1.07,1.59] | 1.30** [1.07,1.59] | 1.30** [1.07,1.59] |
| Lone parent | 2.31*** [1.71,3.11] | 2.31*** [1.71,3.11] | 2.31*** [1.71,3.11] |
| Other no children | 0.99 [0.80,1.24] | 0.99 [0.80,1.24] | 0.99 [0.80,1.24] |
| Other with children | 1.63*** [1.23,2.16] | 1.63*** [1.23,2.16] | 1.63*** [1.23,2.16] |
| Ethnicity | | | |
| White | 1.00 [1.00,1.00] | 1.00 [1.00,1.00] | 1.00 [1.00,1.00] |
| Mixed | 1.90** [1.22,2.96] | 1.90** [1.22,2.96] | 1.90** [1.22,2.96] |
| Asian or Asian British | *1.08 [0.80,1.46]* | *0.98 [0.72,1.35]* | *0.53** [0.35,0.81]* |
| Black or Black British | 1.19 [0.76,1.88] | 1.19 [0.76,1.88] | 1.19 [0.76,1.88] |
| Other ethnic group | 0.94 [0.50,1.79] | 0.94 [0.50,1.79] | 0.94 [0.50,1.79] |
| Annual household income (non equivalised) | | | |
| Less than £19,000 | 1.00 [1.00,1.00] | 1.00 [1.00,1.00] | 1.00 [1.00,1.00] |
| £19,000 - £31,999 | *0.40*** [0.34,0.47]* | *0.35*** [0.30,0.42]* | *0.39*** [0.31,0.49]* |
| £32,000 - £63,999 | *0.16*** [0.14,0.19]* | *0.13*** [0.11,0.16]* | *0.13*** [0.10,0.17]* |
| £64,000 and above | *0.05*** [0.04,0.07]* | *0.03*** [0.02,0.05]* | *0.01*** [0.01,0.03]* |
| Employment status | | | |
| Working | 1.00 [1.00,1.00] | 1.00 [1.00,1.00] | 1.00 [1.00,1.00] |
| Student | 0.46*** [0.31,0.67] | 0.46*** [0.31,0.67] | 0.46*** [0.31,0.67] |
| Retired | 0.38*** [0.32,0.47] | 0.38*** [0.32,0.47] | 0.38*** [0.32,0.47] |
| Unemployed | 1.79** [1.20,2.68] | 1.79** [1.20,2.68] | 1.79** [1.20,2.68] |
| Unable to work due to poor health | 2.13*** [1.58,2.88] | 2.13*** [1.58,2.88] | 2.13*** [1.58,2.88] |
| Homemaker | 1.11 [0.88,1.40] | 1.11 [0.88,1.40] | 1.11 [0.88,1.40] |
| Other | 1.02 [0.76,1.36] | 1.02 [0.76,1.36] | 1.02 [0.76,1.36] |

* p < 0.05, ** p < 0.01, *** p < 0.001

Coefficients that do not satisfy the proportional odds assumption and that have therefore been allowed to vary across different levels of food security have been denoted by italics

Supplementary table S5: Ordinal logistic regression analyses predicting food security status of differing severity, Block 3 showing odds ratios and standard errors, n=17,843

|  | **High vs marginal, low and very low food security** | **High and marginal vs low and very low food security** | **High, marginal and low vs very low food security** |
| --- | --- | --- | --- |
| Survey wave | | | |
| Wave 4 (Oct 2021-Jan 2022) | 1.00 [1.00,1.00] | 1.00 [1.00,1.00] | 1.00 [1.00,1.00] |
| Wave 5 (April - July 2022) | 1.31*** [1.13,1.52] | 1.31*** [1.13,1.52] | 1.31*** [1.13,1.52] |
| Wave 6 (Oct 2022 - Jan 2023) | *1.74*** [1.49,2.03]* | *1.73*** [1.46,2.06]* | *1.82*** [1.46,2.28]* |
| Gender | | | |
| Male | 1.00 [1.00,1.00] | 1.00 [1.00,1.00] | 1.00 [1.00,1.00] |
| Female | 0.92 [0.82,1.03] | 0.92 [0.82,1.03] | 0.92 [0.82,1.03] |
| Age group | | | |
| 16-24 | 1.00 [1.00,1.00] | 1.00 [1.00,1.00] | 1.00 [1.00,1.00] |
| 25-34 | 0.81 [0.60,1.11] | 0.81 [0.60,1.11] | 0.81 [0.60,1.11] |
| 35-44 | 0.66** [0.49,0.90] | 0.66** [0.49,0.90] | 0.66** [0.49,0.90] |
| 45-54 | 0.45*** [0.33,0.61] | 0.45*** [0.33,0.61] | 0.45*** [0.33,0.61] |
| 55-64 | 0.24*** [0.18,0.33] | 0.24*** [0.18,0.33] | 0.24*** [0.18,0.33] |
| 65-74 | 0.22*** [0.16,0.31] | 0.22*** [0.16,0.31] | 0.22*** [0.16,0.31] |
| 75 + | *0.19*** [0.13,0.28]* | *0.14*** [0.09,0.22]* | *0.08*** [0.04,0.16]* |
| Household composition | | | |
| One adult no children | 1.00 [1.00,1.00] | 1.00 [1.00,1.00] | 1.00 [1.00,1.00] |
| Couple no children | 0.93 [0.80,1.09] | 0.93 [0.80,1.09] | 0.93 [0.80,1.09] |
| Couple with children | 1.43*** [1.17,1.75] | 1.43*** [1.17,1.75] | 1.43*** [1.17,1.75] |
| Lone parent | 2.40*** [1.75,3.30] | 2.40*** [1.75,3.30] | 2.40*** [1.75,3.30] |
| Other no children | 1.01 [0.81,1.26] | 1.01 [0.81,1.26] | 1.01 [0.81,1.26] |
| Other with children | 1.73*** [1.31,2.30] | 1.73*** [1.31,2.30] | 1.73*** [1.31,2.30] |
| Ethnicity | | | |
| White | 1.00 [1.00,1.00] | 1.00 [1.00,1.00] | 1.00 [1.00,1.00] |
| Mixed | 1.80** [1.17,2.77] | 1.80** [1.17,2.77] | 1.80** [1.17,2.77] |
| Asian or Asian British | *1.15 [0.85,1.55]* | *1.10 [0.81,1.51]* | *0.62* [0.41,0.94]* |
| Black or Black British | 1.36 [0.86,2.17] | 1.36 [0.86,2.17] | 1.36 [0.86,2.17] |
| Other ethnic group | 0.92 [0.46,1.84] | 0.92 [0.46,1.84] | 0.92 [0.46,1.84] |
| Annual household income (non equivalised) | | | |
| Less than £19,000 | 1.00 [1.00,1.00] | 1.00 [1.00,1.00] | 1.00 [1.00,1.00] |
| £19,000 - £31,999 | *0.40*** [0.34,0.47]* | *0.36*** [0.30,0.43]* | *0.41*** [0.33,0.52]* |
| £32,000 - £63,999 | *0.16*** [0.14,0.19]* | *0.14*** [0.11,0.17]* | *0.14*** [0.10,0.18]* |
| £64,000 and above | *0.05*** [0.04,0.06]* | *0.04*** [0.02,0.05]* | *0.01*** [0.01,0.03]* |
| Employment status | | | |
| Working | 1.00 [1.00,1.00] | 1.00 [1.00,1.00] | 1.00 [1.00,1.00] |
| Student | 0.43*** [0.30,0.63] | 0.43*** [0.30,0.63] | 0.43*** [0.30,0.63] |
| Retired | 0.36*** [0.30,0.44] | 0.36*** [0.30,0.44] | 0.36*** [0.30,0.44] |
| Unemployed | 1.51* [1.03,2.21] | 1.51* [1.03,2.21] | 1.51* [1.03,2.21] |
| Unable to work due to poor health | 1.30 [0.96,1.77] | 1.30 [0.96,1.77] | 1.30 [0.96,1.77] |
| Homemaker | 1.01 [0.81,1.27] | 1.01 [0.81,1.27] | 1.01 [0.81,1.27] |
| Other | 1.01 [0.74,1.38] | 1.01 [0.74,1.38] | 1.01 [0.74,1.38] |
| Long-term health condition status | | | |
| No long-term health condition | 1.00 [1.00,1.00] | 1.00 [1.00,1.00] | 1.00 [1.00,1.00] |
| Has a long-term health condition | *1.74*** [1.52,1.99]* | *2.12*** [1.82,2.48]* | *2.51*** [2.03,3.09]* |
| Food hypersensitivity status | | | |
| No food hypersensitivity | 1.00 [1.00,1.00] | 1.00 [1.00,1.00] | 1.00 [1.00,1.00] |
| Has a food hypersensitivity | 1.19* [1.01,1.40] | 1.17 [0.98,1.40] | 1.49** [1.17,1.90] |

* p < 0.05, ** p < 0.01, *** p < 0.001

Coefficients that do not satisfy the proportional odds assumption and that have therefore been allowed to vary across different levels of food security have been denoted by italics

Supplementary table S6: Logistic regression analyses predicting food security status from interactions with country, showing odds ratios and standard errors, n=13,271

|  | **Country and survey wave** | **Country and gender** | **Country and age group** | **Country and presence of children** | **Country and ethnicity** | **Country and income** | **Country and work status** | **Country and LTHCs** | **Country and hypersensitivity** | **Country and urban-rural status** | **Country and deprivation** |
| --- | --- | --- | --- | --- | --- | --- | --- | --- | --- | --- | --- |
| Intercept | 1.19 [0.83,1.71] | 1.17 [0.82,1.68] | 1.18 [0.82,1.69] | 1.17 [0.82,1.68] | 1.17 [0.82,1.68] | 1.18 [0.82,1.69] | 1.18 [0.82,1.70] | 1.17 [0.81,1.67] | 1.17 [0.82,1.68] | 1.17 [0.82,1.68] | 1.19 [0.83,1.72] |
| Main effects | | | | | | | | | | | |
| England | 1.00 [.,.] | 1.00 [.,.] | 1.00 [.,.] | 1.00 [.,.] | 1.00 [.,.] | 1.00 [.,.] | 1.00 [.,.] | 1.00 [.,.] | 1.00 [.,.] | 1.00 [.,.] | 1.00 [.,.] |
| Wales | 0.98 [0.72,1.35] | 1.24 [0.95,1.62] | 1.25 [0.88,1.76] | 1.23 [0.98,1.54] | 1.25* [1.04,1.49] | 1.19 [0.90,1.57] | 1.08 [0.87,1.34] | 1.34* [1.06,1.70] | 1.26* [1.04,1.53] | 1.36** [1.10,1.68] | 1.09 [0.74,1.62] |
| Northern Ireland | 0.92 [0.69,1.24] | 0.94 [0.73,1.22] | 0.86 [0.63,1.19] | 1.02 [0.82,1.26] | 0.99 [0.83,1.18] | 0.93 [0.72,1.21] | 0.92 [0.75,1.13] | 0.97 [0.78,1.20] | 0.96 [0.79,1.16] | 0.85 [0.69,1.05] | 0.68* [0.47,0.98] |
| Interaction terms | | | | | | | | | | | |
| Country and survey wave | | | | | | | | | | | |
| Wales x wave 4 | 1.00 [.,.] |  |  |  |  |  |  |  |  |  |  |
| Wales x wave 5 | 1.63* [1.07,2.50] |  |  |  |  |  |  |  |  |  |  |
| Wales x wave 6 | 1.11 [0.72,1.71] |  |  |  |  |  |  |  |  |  |  |
| NI x wave 4 | 1.00 [.,.] |  |  |  |  |  |  |  |  |  |  |
| NI x wave 5 | 1.11 [0.74,1.65] |  |  |  |  |  |  |  |  |  |  |
| NI x wave 6 | 1.03 [0.68,1.55] |  |  |  |  |  |  |  |  |  |  |
| Country and gender | | | | | | | | | | | |
| Wales x male |  | 1.00 [.,.] |  |  |  |  |  |  |  |  |  |
| Wales x female |  | 0.98 [0.71,1.36] |  |  |  |  |  |  |  |  |  |
| NI x male |  | 1.00 [.,.] |  |  |  |  |  |  |  |  |  |
| NI x female |  | 1.05 [0.77,1.43] |  |  |  |  |  |  |  |  |  |
| Country and age group | | | | | | | | | | | |
| Wales x 26-34 years |  |  | 1.00 [.,.] |  |  |  |  |  |  |  |  |
| Wales x 35-54 years |  |  | 0.96 [0.63,1.45] |  |  |  |  |  |  |  |  |
| Wales x 55-74 years |  |  | 1.00 [0.64,1.54] |  |  |  |  |  |  |  |  |
| Wales x 75+ years |  |  | 1.08 [0.47,2.45] |  |  |  |  |  |  |  |  |
| NI x 26-34 years |  |  | 1.00 [.,.] |  |  |  |  |  |  |  |  |
| NI x 35-54 years |  |  | 1.16 [0.79,1.71] |  |  |  |  |  |  |  |  |
| NI x 55-74 years |  |  | 1.29 [0.85,1.95] |  |  |  |  |  |  |  |  |
| NI x 75+ years |  |  | 0.91 [0.37,2.24] |  |  |  |  |  |  |  |  |
| Country and presence of children | | | | | | | | | | | |
| Wales x no children |  |  |  | 1.00 [.,.] |  |  |  |  |  |  |  |
| Wales x has children |  |  |  | 0.99 [0.70,1.41] |  |  |  |  |  |  |  |
| NI x no children |  |  |  | 1.00 [.,.] |  |  |  |  |  |  |  |
| NI x has children |  |  |  | 0.87 [0.62,1.22] |  |  |  |  |  |  |  |
| Country and ethnicity | | | | | | | | | | | |
| Wales x White |  |  |  |  | 1.00 [.,.] |  |  |  |  |  |  |
| Wales x ethnic minority |  |  |  |  | 0.77 [0.32,1.86] |  |  |  |  |  |  |
| NI x White |  |  |  |  | 1.00 [.,.] |  |  |  |  |  |  |
| NI x ethnic minority |  |  |  |  | 0.37* [0.15,0.93] |  |  |  |  |  |  |
| Country and income | | | | | | | | | | | |
| Wales x <£19,000 |  |  |  |  |  | 1.00 [.,.] |  |  |  |  |  |
| Wales x £19,000-£31,999 |  |  |  |  |  | 1.22 [0.81,1.83] |  |  |  |  |  |
| Wales x £32,000+ |  |  |  |  |  | 0.92 [0.60,1.40] |  |  |  |  |  |
| NI x <£19,000 |  |  |  |  |  | 1.00 [.,.] |  |  |  |  |  |
| NI x £19,000-£31,999 |  |  |  |  |  | 0.96 [0.65,1.41] |  |  |  |  |  |
| NI x £32,000+ |  |  |  |  |  | 1.18 [0.81,1.72] |  |  |  |  |  |
| Country and working status | | | | | | | | | | | |
| Wales x Working |  |  |  |  |  |  | 1.00 [.,.] |  |  |  |  |
| Wales x Student |  |  |  |  |  |  | 1.45 [0.59,3.55] |  |  |  |  |
| Wales x Retired |  |  |  |  |  |  | 1.44 [0.96,2.15] |  |  |  |  |
| Wales x Unemployed |  |  |  |  |  |  | 1.17 [0.45,3.02] |  |  |  |  |
| Wales x Unable to work due to poor health |  |  |  |  |  |  | 1.80 [0.95,3.40] |  |  |  |  |
| Wales x Homemaker |  |  |  |  |  |  | 0.93 [0.50,1.74] |  |  |  |  |
| Wales x Other |  |  |  |  |  |  | 1.66 [0.72,3.84] |  |  |  |  |
| NI x Working |  |  |  |  |  |  | 1.00 [.,.] |  |  |  |  |
| NI x Student |  |  |  |  |  |  | 1.16 [0.55,2.45] |  |  |  |  |
| NI x Retired |  |  |  |  |  |  | 1.36 [0.90,2.05] |  |  |  |  |
| NI x Unemployed |  |  |  |  |  |  | 0.65 [0.27,1.59] |  |  |  |  |
| NI x Unable to work due to poor health |  |  |  |  |  |  | 1.29 [0.74,2.26] |  |  |  |  |
| NI x Homemaker |  |  |  |  |  |  | 1.12 [0.59,2.11] |  |  |  |  |
| NI x Other |  |  |  |  |  |  | 1.27 [0.56,2.88] |  |  |  |  |
| Country and long-term health condition (LTHC) | | | | | | | | | | | |
| Wales x No LTHC |  |  |  |  |  |  |  | 1.00 [.,.] |  |  |  |
| Wales x Has LTHC |  |  |  |  |  |  |  | 0.79 [0.56,1.11] |  |  |  |
| NI x No LTHC |  |  |  |  |  |  |  | 1.00 [.,.] |  |  |  |
| NI x Has LTHC |  |  |  |  |  |  |  | 1.00 [0.72,1.40] |  |  |  |
| Country and food hypersensitivity | | | | | | | | | | | |
| Wales x No hyper. |  |  |  |  |  |  |  |  | 1.00 [.,.] |  |  |
| Wales x Has hyper. |  |  |  |  |  |  |  |  | 0.83 [0.54,1.29] |  |  |
| NI x No hyper. |  |  |  |  |  |  |  |  | 1.00 [.,.] |  |  |
| NI x Has hyper. |  |  |  |  |  |  |  |  | 1.04 [0.70,1.54] |  |  |
| Country and urban-rural status | | | | | | | | | | | |
| Wales x Urban area |  |  |  |  |  |  |  |  |  | 1.00 [.,.] |  |
| Wales x Rural area |  |  |  |  |  |  |  |  |  | 0.69* [0.47,1.00] |  |
| NI x Urban area |  |  |  |  |  |  |  |  |  | 1.00 [.,.] |  |
| NI x Rural area |  |  |  |  |  |  |  |  |  | 1.34 [0.94,1.91] |  |
| Country and deprivation | | | | | | | | | | | |
| Wales x Decile 1 |  |  |  |  |  |  |  |  |  |  | 1.00 [.,.] |
| Wales x Decile 2 |  |  |  |  |  |  |  |  |  |  | 1.60 [0.94,2.71] |
| Wales x Decile 3 |  |  |  |  |  |  |  |  |  |  | 1.13 [0.66,1.93] |
| Wales x Decile 4 |  |  |  |  |  |  |  |  |  |  | 0.97 [0.55,1.71] |
| Wales x Decile 5 |  |  |  |  |  |  |  |  |  |  | 0.95 [0.54,1.69] |
| NI x Decile 1 |  |  |  |  |  |  |  |  |  |  | 1.00 [.,.] |
| NI x Decile 2 |  |  |  |  |  |  |  |  |  |  | 2.18** [1.33,3.58] |
| NI x Decile 3 |  |  |  |  |  |  |  |  |  |  | 1.17 [0.70,1.97] |
| NI x Decile 4 |  |  |  |  |  |  |  |  |  |  | 1.78* [1.07,2.99] |
| NI x Decile 5 |  |  |  |  |  |  |  |  |  |  | 1.46 [0.87,2.45] |

All models are also adjusted for main effects of covariates, not listed for brevity

Supplementary table S7: Logistic regression analyses predicting emergency food receipt from interactions with country, showing odds ratios and standard errors, n=8,730

|  | **Country and survey wave** | **Country and gender** | **Country and FI status** | **Country and age group** | **Country and presence of children** | **Country and ethnicity** | **Country and income** | **Country and work status** | **Country and LTHCs** | **Country and hypersensitivity** | **Country and urban-rural status** | **Country and deprivation** |
| --- | --- | --- | --- | --- | --- | --- | --- | --- | --- | --- | --- | --- |
| Intercept | 0.09*** [0.03,0.24] | 0.09*** [0.03,0.23] | 0.09*** [0.03,0.25] | 0.09*** [0.03,0.24] | 0.09*** [0.03,0.25] | 0.09*** [0.03,0.23] | 0.09*** [0.03,0.24] | 0.08*** [0.03,0.23] | 0.09*** [0.03,0.24] | 0.09*** [0.03,0.23] | 0.09*** [0.03,0.24] | 0.09*** [0.03,0.24] |
| Main effects | | | | | | | | | | | | |
| England | 1.000 [.,.] | 1.000 [.,.] | 1.000 [.,.] | 1.000 [.,.] | 1.000 [.,.] | 1.000 [.,.] | 1.000 [.,.] | 1.000 [.,.] | 1.000 [.,.] | 1.000 [.,.] | 1.000 [.,.] | 1.000 [.,.] |
| Wales | 0.50 [0.23,1.10] | 0.97 [0.46,2.04] | 0.36 [0.11,1.24] | 0.77 [0.33,1.81] | 0.89 [0.49,1.60] | 0.89 [0.54,1.47] | 0.72 [0.41,1.29] | 1.34 [0.74,2.43] | 0.83 [0.37,1.86] | 0.81 [0.48,1.39] | 0.79 [0.45,1.39] | 0.46 [0.21,1.02] |
| Northern Ireland | 0.98 [0.43,2.24] | 1.52 [0.77,3.00] | 0.59 [0.26,1.38] | 1.06 [0.51,2.21] | 1.53 [0.85,2.74] | 1.26 [0.79,2.03] | 0.93 [0.53,1.64] | 1.60 [0.89,2.88] | 1.07 [0.54,2.12] | 1.18 [0.72,1.93] | 1.09 [0.62,1.93] | 0.66 [0.30,1.46] |
| Interaction terms | | | | | | | | | | | | |
| Country and survey wave | | | | | | | | | | | | |
| Wales x wave 4 | 1.00 [.,.] |  |  |  |  |  |  |  |  |  |  |  |
| Wales x wave 5 | 2.62 [0.80,8.58] |  |  |  |  |  |  |  |  |  |  |  |
| Wales x wave 6 | 1.95 [0.69,5.52] |  |  |  |  |  |  |  |  |  |  |  |
| NI x wave 4 | 1.00 [.,.] |  |  |  |  |  |  |  |  |  |  |  |
| NI x wave 5 | 1.97 [0.70,5.54] |  |  |  |  |  |  |  |  |  |  |  |
| NI x wave 6 | 1.02 [0.35,2.99] |  |  |  |  |  |  |  |  |  |  |  |
| Country and gender | | | | | | | | | | | | |
| Wales x male |  | 1.00 [.,.] |  |  |  |  |  |  |  |  |  |  |
| Wales x female |  | 0.80 [0.33,1.92] |  |  |  |  |  |  |  |  |  |  |
| NI x male |  | 1.00 [.,.] |  |  |  |  |  |  |  |  |  |  |
| NI x female |  | 0.69 [0.31,1.51] |  |  |  |  |  |  |  |  |  |  |
| Country and food security status | | | | | | | | | | | | |
| Wales x food secure |  |  | 1.00 [.,.] |  |  |  |  |  |  |  |  |  |
| Wales x food insecure |  |  | 2.91 [0.78,10.93] |  |  |  |  |  |  |  |  |  |
| NI x food secure |  |  | 1.00 [.,.] |  |  |  |  |  |  |  |  |  |
| NI x food insecure |  |  | 2.65* [1.02,6.93] |  |  |  |  |  |  |  |  |  |
| Country and age group | | | | | | | | | | | | |
| Wales x 26-34 years |  |  |  | 1.00 [.,.] |  |  |  |  |  |  |  |  |
| Wales x 35-54 years |  |  |  | 1.61 [0.61,4.23] |  |  |  |  |  |  |  |  |
| Wales x 55-74 years |  |  |  | 0.41 [0.09,1.84] |  |  |  |  |  |  |  |  |
| Wales x 75+ years |  |  |  | 1.00 [.,.] |  |  |  |  |  |  |  |  |
| NI x 26-34 years |  |  |  | 1.00 [.,.] |  |  |  |  |  |  |  |  |
| NI x 35-54 years |  |  |  | 1.04 [0.41,2.64] |  |  |  |  |  |  |  |  |
| NI x 55-74 years |  |  |  | 1.89 [0.64,5.57] |  |  |  |  |  |  |  |  |
| NI x 75+ years |  |  |  | 0.60 [0.05,8.00] |  |  |  |  |  |  |  |  |
| Country and presence of children | | | | | | | | | | | | |
| Wales x no children |  |  |  |  | 1.00 [.,.] |  |  |  |  |  |  |  |
| Wales x has children |  |  |  |  | 0.91 [0.35,2.40] |  |  |  |  |  |  |  |
| NI x no children |  |  |  |  | 1.00 [.,.] |  |  |  |  |  |  |  |
| NI x has children |  |  |  |  | 0.61 [0.27,1.38] |  |  |  |  |  |  |  |
| Country and ethnicity | | | | | | | | | | | | |
| Wales x White |  |  |  |  |  | 1.00 [.,.] |  |  |  |  |  |  |
| Wales x ethnic minority |  |  |  |  |  | 0.64 [0.11,3.76] |  |  |  |  |  |  |
| NI x White |  |  |  |  |  | 1.00 [.,.] |  |  |  |  |  |  |
| NI x ethnic minority |  |  |  |  |  | 0.51 [0.08,3.35] |  |  |  |  |  |  |
| Country and income | | | | | | | | | | | | |
| Wales x <£19,000 |  |  |  |  |  |  | 1.00 [.,.] |  |  |  |  |  |
| Wales x £19,000-£31,999 |  |  |  |  |  |  | 1.76 [0.61,5.10] |  |  |  |  |  |
| Wales x £32,000+ |  |  |  |  |  |  | 2.42 [0.53,10.92] |  |  |  |  |  |
| NI x <£19,000 |  |  |  |  |  |  | 1.00 [.,.] |  |  |  |  |  |
| NI x £19,000-£31,999 |  |  |  |  |  |  | 1.91 [0.81,4.47] |  |  |  |  |  |
| NI x £32,000+ |  |  |  |  |  |  | 4.57* [1.22,17.06] |  |  |  |  |  |
| Country and working status | | | | | | | | | | | | |
| Wales x Working |  |  |  |  |  |  |  | 1.00 [.,.] |  |  |  |  |
| Wales x Student |  |  |  |  |  |  |  | 1.01 [0.23,4.47] |  |  |  |  |
| Wales x Retired |  |  |  |  |  |  |  | 0.15* [0.03,0.89] |  |  |  |  |
| Wales x Unemployed |  |  |  |  |  |  |  | 0.17* [0.04,0.74] |  |  |  |  |
| Wales x Sick |  |  |  |  |  |  |  | 0.59 [0.20,1.72] |  |  |  |  |
| Wales x Homemaker |  |  |  |  |  |  |  | 0.40 [0.11,1.48] |  |  |  |  |
| Wales x Other |  |  |  |  |  |  |  | 0.54 [0.09,3.33] |  |  |  |  |
| NI x Working |  |  |  |  |  |  |  | 1.00 [.,.] |  |  |  |  |
| NI x Student |  |  |  |  |  |  |  | 0.49 [0.11,2.25] |  |  |  |  |
| NI x Retired |  |  |  |  |  |  |  | 1.82 [0.52,6.42] |  |  |  |  |
| NI x Unemployed |  |  |  |  |  |  |  | 0.33 [0.09,1.22] |  |  |  |  |
| NI x Sick |  |  |  |  |  |  |  | 0.29 [0.08,1.13] |  |  |  |  |
| NI x Homemaker |  |  |  |  |  |  |  | 0.94 [0.27,3.27] |  |  |  |  |
| NI x Other |  |  |  |  |  |  |  | 1.00 [.,.] |  |  |  |  |
| Country and long-term health condition (LTHC) | | | | | | | | | | | | |
| Wales x No LTHC |  |  |  |  |  |  |  |  | 1.00 [.,.] |  |  |  |
| Wales x Has LTHC |  |  |  |  |  |  |  |  | 1.05 [0.38,2.93] |  |  |  |
| NI x No LTHC |  |  |  |  |  |  |  |  | 1.00 [.,.] |  |  |  |
| NI x Has LTHC |  |  |  |  |  |  |  |  | 1.36 [0.55,3.36] |  |  |  |
| Country and food hypersensitivity | | | | | | | | | | | | |
| Wales x No hyper. |  |  |  |  |  |  |  |  |  | 1.00 [.,.] |  |  |
| Wales x Has hyper. |  |  |  |  |  |  |  |  |  | 1.32 [0.41,4.22] |  |  |
| NI x No hyper. |  |  |  |  |  |  |  |  |  | 1.00 [.,.] |  |  |
| NI x Has hyper. |  |  |  |  |  |  |  |  |  | 1.27 [0.46,3.48] |  |  |
| Country and urban-rural status | | | | | | | | | | | | |
| Wales x Urban area |  |  |  |  |  |  |  |  |  |  | 1.00 [.,.] |  |
| Wales x Rural area |  |  |  |  |  |  |  |  |  |  | 1.41 [0.46,4.28] |  |
| NI x Urban area |  |  |  |  |  |  |  |  |  |  | 1.00 [.,.] |  |
| NI x Rural area |  |  |  |  |  |  |  |  |  |  | 1.41 [0.53,3.76] |  |
| Country and deprivation | | | | | | | | | | | | |
| Wales x Decile 1 |  |  |  |  |  |  |  |  |  |  |  | 1.00 [.,.] |
| Wales x Decile 2 |  |  |  |  |  |  |  |  |  |  |  | 5.78** [1.92,17.38] |
| Wales x Decile 3 |  |  |  |  |  |  |  |  |  |  |  | 3.05 [0.84,11.09] |
| Wales x Decile 4 |  |  |  |  |  |  |  |  |  |  |  | 1.73 [0.33,9.18] |
| Wales x Decile 5 |  |  |  |  |  |  |  |  |  |  |  | 0.95 [0.18,4.98] |
| NI x Decile 1 |  |  |  |  |  |  |  |  |  |  |  | 1.00 [.,.] |
| NI x Decile 2 |  |  |  |  |  |  |  |  |  |  |  | 5.05** [1.67,15.31] |
| NI x Decile 3 |  |  |  |  |  |  |  |  |  |  |  | 1.19 [0.32,4.44] |
| NI x Decile 4 |  |  |  |  |  |  |  |  |  |  |  | 3.23 [0.89,11.78] |
| NI x Decile 5 |  |  |  |  |  |  |  |  |  |  |  | 0.90 [0.21,3.82] |

All models are also adjusted for main effects of covariates, not listed for brevity

Supplementary table S8: Logistic regression analyses predicting emergency food receipt from very low food security and other predictors, showing odds ratios and standard errors, n=11,161

|  | **Bivariate associations** | **Block 1: Demographics** | **Block 2: Demographics plus financial characteristics** | **Block 3: Demographics plus financial characteristics plus health** | **Block 4: Demographics plus financial characteristics plus health**  **plus local characteristics** |
| --- | --- | --- | --- | --- | --- |
| Intercept | Variable, from 0.01 to 0.12 | 0.07*** [0.03,0.16] | 0.11*** [0.04,0.27] | 0.10*** [0.04,0.26] | 0.14*** [0.05,0.37] |
| Survey wave | | | | | |
| Wave 4 (Oct 2021-Jan 2022) | 1.00  [.,.] | 1.00  [.,.] | 1.00  [.,.] | 1.00  [.,.] | 1.00  [.,.] |
| Wave 5 (April - July 2022) | 0.72 [0.46,1.12] | 0.59* [0.36,0.95] | 0.62 [0.38,1.03] | 0.62 [0.37,1.04] | 0.62 [0.38,1.02] |
| Wave 6 (Oct 2022 - Jan 2023) | 0.85 [0.53,1.38] | 0.58* [0.34,0.97] | 0.65 [0.38,1.11] | 0.66 [0.38,1.13] | 0.68 [0.40,1.15] |
| Food security status | | | | | |
| High, marginal or low food security | 1.00  [.,.] | 1.00  [.,.] | 1.00  [.,.] | 1.00  [.,.] | 1.00  [.,.] |
| Very low food security | 15.02*** [10.16,22.21] | 13.45*** [9.13,19.80] | 5.56*** [3.59,8.61] | 5.12*** [3.33,7.87] | 5.20*** [3.40,7.94] |
| Gender | | | | | |
| Male | 1.00  [.,.] | 1.00  [.,.] | 1.00  [.,.] | 1.00  [.,.] | 1.00  [.,.] |
| Female | 1.08 [0.76,1.54] | 1.06 [0.73,1.55] | 0.94 [0.63,1.40] | 0.92 [0.61,1.38] | 0.94 [0.63,1.41] |
| Age group | | | | | |
| 16-24 | 1.00  [.,.] | 1.00  [.,.] | 1.00  [.,.] | 1.00  [.,.] | 1.00  [.,.] |
| 25-34 | 0.42** [0.24,0.76] | 0.37** [0.19,0.71] | 0.38* [0.18,0.81] | 0.38* [0.18,0.81] | 0.36** [0.17,0.76] |
| 35-44 | 0.51* [0.30,0.89] | 0.53* [0.28,0.99] | 0.58 [0.28,1.17] | 0.57 [0.28,1.19] | 0.57 [0.28,1.16] |
| 45-54 | 0.33*** [0.19,0.59] | 0.44** [0.24,0.82] | 0.46* [0.21,1.00] | 0.46 [0.21,1.01] | 0.48 [0.22,1.05] |
| 55-64 | 0.22*** [0.11,0.42] | 0.36** [0.18,0.73] | 0.33** [0.15,0.74] | 0.32** [0.14,0.73] | 0.36* [0.16,0.82] |
| 65-74 | 0.10*** [0.04,0.23] | 0.22** [0.09,0.55] | 0.37 [0.10,1.37] | 0.36 [0.10,1.34] | 0.38 [0.10,1.42] |
| 75 + | 0.20* [0.05,0.75] | 0.39 [0.09,1.72] | 0.84 [0.15,4.51] | 0.75 [0.13,4.21] | 0.87 [0.16,4.78] |
| Household composition | | | | | |
| One adult no children | 1.00  [.,.] | 1.00  [.,.] | 1.00  [.,.] | 1.00  [.,.] | 1.00  [.,.] |
| Couple no children | 0.54* [0.32,0.89] | 0.62 [0.35,1.10] | 0.91 [0.52,1.60] | 0.92 [0.52,1.62] | 0.91 [0.52,1.58] |
| Couple with children | 1.09 [0.65,1.86] | 0.72 [0.37,1.40] | 1.23 [0.62,2.43] | 1.27 [0.64,2.54] | 1.36 [0.68,2.71] |
| Lone parent | 4.20*** [2.26,7.80] | 1.60 [0.72,3.58] | 1.77 [0.75,4.16] | 1.81 [0.76,4.31] | 1.94 [0.82,4.57] |
| Other no children | 0.84 [0.43,1.67] | 0.54 [0.26,1.14] | 0.91 [0.43,1.90] | 0.90 [0.43,1.90] | 0.94 [0.47,1.89] |
| Other with children | 1.09 [0.51,2.30] | 0.43 [0.18,1.05] | 0.72 [0.31,1.70] | 0.71 [0.30,1.68] | 0.72 [0.30,1.71] |
| Ethnicity | | | | | |
| White | 1.00  [.,.] | 1.00  [.,.] | 1.00  [.,.] | 1.00  [.,.] | 1.00  [.,.] |
| Ethnic minority | 2.39** [1.41,4.05] | 2.42** [1.34,4.37] | 1.87* [1.03,3.40] | 1.95* [1.07,3.55] | 1.83 [0.97,3.46] |
| Household income | | | | | |
| Less than £19,000 | 1.00  [.,.] |  | 1.00  [.,.] | 1.00  [.,.] | 1.00  [.,.] |
| £19,000 - £31,999 | 0.22*** [0.13,0.37] |  | 0.40** [0.22,0.72] | 0.40** [0.22,0.72] | 0.42** [0.23,0.76] |
| £32,000 and above | 0.05*** [0.03,0.12] |  | 0.14*** [0.05,0.36] | 0.14*** [0.05,0.36] | 0.16*** [0.06,0.42] |
| Employment status | | | | | |
| Working | 1.00  [.,.] |  | 1.00  [.,.] | 1.00  [.,.] | 1.00  [.,.] |
| Student | 3.89*** [1.90,7.96] |  | 1.03 [0.38,2.78] | 1.00 [0.36,2.79] | 1.09 [0.40,3.00] |
| Retired | 0.47 [0.21,1.05] |  | 0.47 [0.16,1.37] | 0.45 [0.15,1.34] | 0.49 [0.16,1.50] |
| Unemployed | 12.86*** [7.38,22.38] |  | 3.16*** [1.69,5.92] | 2.91** [1.52,5.54] | 3.10*** [1.61,5.95] |
| Unable to work due to poor health | 13.77*** [8.54,22.22] |  | 4.04*** [2.31,7.06] | 3.21*** [1.67,6.18] | 3.26*** [1.67,6.36] |
| Homemaker | 4.48*** [2.59,7.74] |  | 1.94 [0.92,4.11] | 1.82 [0.85,3.91] | 1.87 [0.86,4.06] |
| Other | 4.47*** [2.28,8.80] |  | 2.53* [1.20,5.31] | 2.49* [1.19,5.22] | 2.44* [1.10,5.40] |
| Long-term health condition status | | | | | |
| No long-term health condition | 1.00  [.,.] |  |  | 1.00  [.,.] | 1.00  [.,.] |
| Has a long-term health condition | 2.92*** [1.98,4.31] |  |  | 1.47 [0.89,2.44] | 1.40 [0.84,2.32] |
| Food hypersensitivity status | | | | | |
| No food hypersensitivity | 1.00  [.,.] |  |  | 1.00  [.,.] | 1.00  [.,.] |
| Has a food hypersensitivity | 1.18 [0.74,1.88] |  |  | 0.95 [0.54,1.68] | 0.97 [0.56,1.68] |
| Urban/rural classification | | | | | |
| Urban | 1.00  [.,.] |  |  |  | 1.00  [.,.] |
| Rural | 0.55* [0.32,0.95] |  |  |  | 1.15 [0.65,2.05] |
| Country | | | | | |
| England | 1.00  [.,.] |  |  |  | 1.00  [.,.] |
| Wales | 1.06 [0.74,1.50] |  |  |  | 0.91 [0.60,1.37] |
| Northern Ireland | 1.27 [0.92,1.76] |  |  |  | 1.43 [0.96,2.14] |
| Index of Multiple Deprivation (IMD) | | | | | |
| Quintile 1 (most deprived) | 1.00  [.,.] |  |  |  | 1.00  [.,.] |
| Quintile 2 | 0.37*** [0.24,0.58] |  |  |  | 0.43** [0.26,0.72] |
| Quintile 3 | 0.34*** [0.19,0.60] |  |  |  | 0.55 [0.28,1.07] |
| Quintile 4 | 0.22*** [0.10,0.48] |  |  |  | 0.52 [0.22,1.21] |
| Quintile 5 (least deprived) | 0.11*** [0.05,0.24] |  |  |  | 0.33** [0.14,0.75] |

* p < 0.05, ** p < 0.01, *** p < 0.001

Supplementary table S9: Logistic regression analyses predicting emergency food receipt, without controlling for food security status, showing odds ratios and standard errors, n=11,161

|  | **Bivariate associations** | **Block 1: Demographics** | **Block 2: Demographics plus financial characteristics** | **Block 3: Demographics plus financial characteristics plus health** | **Block 4: Demographics plus financial characteristics plus health**  **plus local characteristics** |
| --- | --- | --- | --- | --- | --- |
| Intercept | Variable, from 0.01 to 0.12 | 0.19*** [0.09,0.39] | 0.22** [0.08,0.59] | 0.17*** [0.06,0.47] | 0.24** [0.08,0.69] |
| Survey wave | | | | | |
| Wave 4 (Oct 2021-Jan 2022) | 1.00  [.,.] | 1.00  [.,.] | 1.00  [.,.] | 1.00  [.,.] | 1.00  [.,.] |
| Wave 5 (April - July 2022) | 0.72 [0.46,1.12] | 0.71 [0.45,1.12] | 0.74 [0.46,1.19] | 0.73 [0.45,1.20] | 0.75 [0.47,1.21] |
| Wave 6 (Oct 2022 - Jan 2023) | 0.85 [0.53,1.38] | 0.80 [0.50,1.29] | 0.78 [0.46,1.31] | 0.79 [0.46,1.33] | 0.82 [0.49,1.38] |
| Gender | | | | | |
| Male | 1.00  [.,.] | 1.00  [.,.] | 1.00  [.,.] | 1.00  [.,.] | 1.00  [.,.] |
| Female | 1.08 [0.76,1.54] | 1.09 [0.76,1.55] | 0.90 [0.61,1.33] | 0.86 [0.57,1.30] | 0.87 [0.59,1.31] |
| Age group | | | | | |
| 16-24 | 1.00  [.,.] | 1.00  [.,.] | 1.00  [.,.] | 1.00  [.,.] | 1.00  [.,.] |
| 25-34 | 0.42** [0.24,0.76] | 0.37** [0.20,0.68] | 0.39* [0.18,0.84] | 0.38* [0.17,0.83] | 0.36* [0.16,0.79] |
| 35-44 | 0.51* [0.30,0.89] | 0.41** [0.23,0.73] | 0.53 [0.25,1.11] | 0.52 [0.24,1.12] | 0.52 [0.24,1.11] |
| 45-54 | 0.33*** [0.19,0.59] | 0.31*** [0.18,0.54] | 0.38* [0.17,0.83] | 0.39* [0.17,0.87] | 0.41* [0.18,0.91] |
| 55-64 | 0.22*** [0.11,0.42] | 0.23*** [0.12,0.42] | 0.24*** [0.10,0.54] | 0.23*** [0.10,0.54] | 0.26** [0.11,0.60] |
| 65-74 | 0.10*** [0.04,0.23] | 0.10*** [0.04,0.23] | 0.21* [0.06,0.74] | 0.20* [0.06,0.74] | 0.22* [0.06,0.80] |
| 75 + | 0.20* [0.05,0.75] | 0.18* [0.05,0.72] | 0.52 [0.10,2.64] | 0.44 [0.08,2.40] | 0.51 [0.10,2.71] |
| Household composition | | | | | |
| One adult no children | 1.00  [.,.] | 1.00  [.,.] | 1.00  [.,.] | 1.00  [.,.] | 1.00  [.,.] |
| Couple no children | 0.54* [0.32,0.89] | 0.49** [0.29,0.83] | 0.95 [0.54,1.65] | 0.95 [0.54,1.66] | 0.95 [0.54,1.65] |
| Couple with children | 1.09 [0.65,1.86] | 0.64 [0.34,1.21] | 1.27 [0.66,2.44] | 1.40 [0.73,2.71] | 1.50 [0.78,2.90] |
| Lone parent | 4.20*** [2.26,7.80] | 2.57** [1.31,5.08] | 2.50* [1.18,5.28] | 2.51* [1.16,5.44] | 2.66* [1.23,5.75] |
| Other no children | 0.84 [0.43,1.67] | 0.43* [0.21,0.87] | 0.89 [0.43,1.83] | 0.89 [0.43,1.83] | 0.93 [0.46,1.87] |
| Other with children | 1.09 [0.51,2.30] | 0.43* [0.19,0.95] | 0.89 [0.40,1.97] | 0.87 [0.39,1.97] | 0.90 [0.39,2.05] |
| Ethnicity | | | | | |
| White | 1.00  [.,.] | 1.00  [.,.] | 1.00  [.,.] | 1.00  [.,.] | 1.00  [.,.] |
| Ethnic minority | 2.39** [1.41,4.05] | 1.81* [1.06,3.06] | 1.43 [0.82,2.48] | 1.58 [0.90,2.78] | 1.47 [0.80,2.70] |
| Household income | | | | | |
| Less than £19,000 | 1.00  [.,.] |  | 1.00  [.,.] | 1.00  [.,.] | 1.00  [.,.] |
| £19,000 - £31,999 | 0.22*** [0.13,0.37] |  | 0.31*** [0.18,0.55] | 0.33*** [0.19,0.57] | 0.35*** [0.20,0.60] |
| £32,000 and above | 0.05*** [0.03,0.12] |  | 0.08*** [0.03,0.19] | 0.08*** [0.03,0.21] | 0.10*** [0.04,0.24] |
| Employment status | | | | | |
| Working | 1.00  [.,.] |  | 1.00  [.,.] | 1.00  [.,.] | 1.00  [.,.] |
| Student | 3.89*** [1.90,7.96] |  | 0.83 [0.30,2.26] | 0.81 [0.29,2.31] | 0.88 [0.31,2.49] |
| Retired | 0.47 [0.21,1.05] |  | 0.41 [0.16,1.10] | 0.39 [0.14,1.08] | 0.42 [0.15,1.19] |
| Unemployed | 12.86*** [7.38,22.38] |  | 4.45*** [2.39,8.28] | 3.81*** [2.03,7.14] | 4.11*** [2.20,7.68] |
| Unable to work due to poor health | 13.77*** [8.54,22.22] |  | 5.43*** [3.14,9.38] | 3.47*** [1.84,6.52] | 3.52*** [1.85,6.69] |
| Homemaker | 4.48*** [2.59,7.74] |  | 2.21* [1.11,4.40] | 1.92 [0.94,3.92] | 1.99 [0.97,4.06] |
| Other | 4.47*** [2.28,8.80] |  | 2.83** [1.37,5.85] | 2.75** [1.34,5.67] | 2.65* [1.22,5.75] |
| Long-term health condition status | | | | | |
| No long-term health condition | 1.00  [.,.] |  |  | 1.00  [.,.] | 1.00  [.,.] |
| Has a long-term health condition | 2.92*** [1.98,4.31] |  |  | 2.05** [1.25,3.38] | 1.92* [1.16,3.18] |
| Food hypersensitivity status | | | | | |
| No food hypersensitivity | 1.00  [.,.] |  |  | 1.00  [.,.] | 1.00  [.,.] |
| Has a food hypersensitivity | 1.18 [0.74,1.88] |  |  | 1.03 [0.58,1.83] | 1.06 [0.60,1.86] |
| Urban/rural classification | | | | | |
| Urban | 1.00  [.,.] |  |  |  | 1.00  [.,.] |
| Rural | 0.55* [0.32,0.95] |  |  |  | 1.16 [0.67,2.03] |
| Country | | | | | |
| England | 1.00  [.,.] |  |  |  | 1.00  [.,.] |
| Wales | 1.06 [0.74,1.50] |  |  |  | 0.97 [0.65,1.43] |
| Northern Ireland | 1.27 [0.92,1.76] |  |  |  | 1.31 [0.88,1.95] |
| Index of Multiple Deprivation (IMD) | | | | | |
| Quintile 1 (most deprived) | 1.00  [.,.] |  |  |  | 1.00  [.,.] |
| Quintile 2 | 0.37*** [0.24,0.58] |  |  |  | 0.45** [0.27,0.75] |
| Quintile 3 | 0.34*** [0.19,0.60] |  |  |  | 0.58 [0.31,1.08] |
| Quintile 4 | 0.22*** [0.10,0.48] |  |  |  | 0.50 [0.22,1.14] |
| Quintile 5 (least deprived) | 0.11*** [0.05,0.24] |  |  |  | 0.33** [0.15,0.75] |

* p < 0.05, ** p < 0.01, *** p < 0.001
