# Supplementary material for: Food insecurity and food bank use: who is most at risk of severe food insecurity and who uses food banks?
Source: Public Health Nutr. 2024 Sep 26;27(1):e174. doi: 10.1017/S1368980024001393 (PMC11505125; doi:10.1017/S1368980024001393)
Supplement: Garratt and Armstrong supplementary material 2 — Garratt and Armstrong supplementary material [file S1368980024001393sup002.pdf]

Supplementary materials

Supplementary table S1: Weighted bivariate associations between food security status and predictor variables, pooling waves 4 to 6 (n=16,659)

	**High food security**<br><br>**n=11,016**	**Marginal food security**<br><br>**n=2,184**	**Low food security**<br><br>**n=1,826**	**Very low food security**<br><br>**n=1,632**	**p value from chi-squared test of association**
Survey wave					
Wave 4 (Oct 2021 - Jan 2022)	70.4%	12.0%	10.3%	7.3%	
Wave 5 (April - July 2022)	66.6%	13.3%	10.4%	9.7%	
Wave 6 (Oct 2022 - Jan 2023)	61.4%	14.0%	12.2%	12.4%	**<0.001**
Gender					
Male	67.5%	12.5%	10.4%	9.7%	
Female	65.4%	13.6%	11.3%	9.7%	**<0.001**
Age group					
16-24	46.2%	18.3%	17.3%	18.3%	
25-34	54.0%	16.0%	14.9%	15.1%	
35-44	57.6%	16.0%	14.7%	11.7%	
45-54	67.6%	12.2%	9.9%	10.3%	
55-64	76.6%	9.3%	7.8%	6.3%	
65-74	81.8%	10.0%	5.4%	2.7%	
75 +	84.9%	9.1%	4.8%	1.3%	**<0.001**
Household composition					
One adult no children	68.5%	13.0%	8.4%	10.1%	
Couple no children	74.6%	10.9%	8.5%	6.1%	
Couple with children	56.6%	16.2%	14.4%	12.8%	**<0.001**

Lone parent	29.9%	16.4%	19.2%	34.5%	
Other no children	67.8%	13.0%	10.5%	8.7%	
Other with children	52.6%	15.5%	16.5%	15.4%	
Ethnicity					
White	69.3%	12.2%	9.0%	9.5%	
Mixed	43.7%	15.5%	24.3%	16.6%	
Asian or Asian British	51.2%	17.7%	21.0%	10.2%	
Black or Black British	48.0%	22.5%	19.9%	9.5%	
Other ethnic group	53.8%	15.8%	17.3%	13.1%	**<0.001**
Annual household income (non equivalised)					
Less than £19,000	42.9%	13.9%	19.8%	23.4%	
£19,000 - £31,999	59.6%	15.6%	12.6%	12.3%	
£32,000 - £63,999	73.4%	13.3%	8.4%	4.8%	
£64,000 and above	89.8%	6.5%	3.0%	0.6%	**<0.001**
Employment status					
Working	65.8%	13.7%	11.5%	9.0%	
Student	49.4%	19.2%	15.9%	15.5%	
Retired	84.7%	9.0%	4.4%	1.9%	
Unemployed	30.5%	11.2%	20.3%	38.0%	
Unable to work due to poor health	29.4%	14.3%	20.0%	36.3%	
Homemaker	50.4%	14.4%	16.9%	18.3%	
Other	56.6%	14.4%	14.1%	15.0%	**<0.001**
Long-term health condition status					

No long-term health condition	70.3%	13.4%	10.1%	6.2%	
Has a long-term health condition	59.3%	11.5%	12.5%	16.8%	**<0.001**
Food hypersensitivity status					
No food hypersensitivity	67.4%	12.8%	11.0%	8.8%	
Has a food hypersensitivity	63.3%	14.1%	9.5%	13.2%	**<0.001**
Urban/rural classification					
Urban	63.6%	13.9%	12.0%	10.6%	
Rural	76.1%	10.1%	7.0%	6.8%	**<0.001**
Country					
England	66.4%	13.1%	10.9%	9.6%	
Wales	62.9%	13.6%	11.2%	12.4%	
Northern Ireland	63.9%	14.2%	11.8%	10.1%	**<0.001**
Region					
North-East England	64.6%	13.5%	11.2%	10.7%	
North-West England	65.1%	13.3%	12.3%	9.3%	
Yorkshire and the Humber	62.6%	12.4%	11.0%	14.0%	
West Midlands	61.2%	15.7%	11.2%	12.0%	
East Midlands	64.5%	13.7%	11.2%	10.6%	
East of England	71.4%	11.4%	9.5%	7.7%	
South-East England	70.4%	11.7%	9.2%	8.7%	
South-West England	71.8%	11.8%	7.8%	8.6%	
Greater London	63.6%	14.4%	14.2%	7.8%	
Wales	62.9%	13.6%	11.2%	12.4%	**<0.001**

Northern Ireland	63.9%	14.2%	11.8%	10.1%	
Multiple deprivation					
Quintile 1 - Most deprived	47.4%	17.4%	17.8%	17.4%	
Quintile 2	63.5%	14.3%	11.5%	10.7%	
Quintile 3	66.8%	11.7%	11.5%	10.0%	
Quintile 4	73.1%	12.0%	8.0%	6.9%	
Quintile 5 - Least deprived	79.3%	10.2%	6.3%	4.2%	**<0.0 01**

Significant associations as determined by the chi-squared test of association are denoted in bold text

Supplementary table S2: Weighted bivariate associations between emergency food receipt in the past 12 months and predictor variables, pooling waves 4 to 6 (n=10,913)

	**Received emergency food (%)**	**Not received emergency food (%)**	**p value from chi-squared test of association**
	**n=395**	**n=10,518**	
Survey wave			
Wave 4 (Oct 2021-Jan 2022)	4.2%	95.8%	
Wave 5 (April - July 2022)	3.0%	97.0%	
Wave 6 (Oct 2022 - Jan 2023)	3.7%	96.3%	0.636
Food security status			
High	0.7%	99.3%	
Marginal	3.7%	96.4%	
Low	7.5%	92.5%	
Very low	20.6%	79.4%	**<0.001**
Gender			
Male	3.5%	96.6%	
Female	3.6%	96.4%	0.085
Age group			
16-24	8.2%	91.8%	
25-34	3.6%	96.4%	
35-44	4.7%	95.3%	
45-54	3.9%	96.1%	
55-64	2.2%	97.8%	
65-74	1.4%	98.7%	
75 +	1.3%	98.7%	**<0.001**

Household composition			
One adult no children	4.4%	95.7%	
Couple no children	2.2%	97.8%	
Couple with children	4.8%	95.2%	
Lone parent	14.7%	85.3%	
Other no children	3.7%	96.3%	
Other with children	4.5%	95.5%	**<0.001**
Ethnicity			
White	2.9%	97.1%	
Mixed	9.8%	90.2%	
Asian or Asian British	7.8%	92.2%	
Black or Black British	11.0%	89.0%	
Other ethnic group	3.2%	96.8%	**<0.001**
Annual household income (non equivalised)			
Less than £19,000	11.6%	88.4%	
£19,000 - £31,999	2.7%	97.3%	
£32,000 and above	0.3%	99.7%	**<0.001**
Employment status			
Working	2.2%	97.9%	
Student	7.5%	92.5%	
Retired	1.0%	99.0%	
Unemployed	19.0%	81.0%	
Unable to work due to poor health	20.6%	79.5%	**<0.001**

Homemaker	9.4%	90.6%	
Other	7.5%	92.5%	
Long-term health condition status			
No long-term health condition	2.2%	97.8%	
Has a long-term health condition	6.4%	93.6%	**<0.001**
Food hypersensitivity status			
No food hypersensitivity	3.5%	96.5%	
Has a food hypersensitivity	4.0%	96.0%	**0.015**
Urban/rural classification			
Urban	4.0%	96.0%	
Rural	2.3%	97.7%	**0.001**
Country			
England	3.6%	96.4%	
Wales	3.8%	96.2%	
Northern Ireland	4.3%	95.7%	**0.044**
Region			
North-East England	3.1%	96.9%	
North-West England	3.9%	96.1%	
Yorkshire and the Humber	4.6%	95.4%	
West Midlands	3.9%	96.1%	
East Midlands	4.5%	95.5%	
East of England	3.2%	96.8%	
South-East England	2.2%	97.8%	**0.002**

South-West England	2.9%	97.1%	
Greater London	4.3%	95.7%	
Wales	3.8%	96.2%	
Northern Ireland	4.3%	95.7%	
Multiple deprivation			
Quintile 1 - Most deprived	8.1%	91.9%	
Quintile 2	3.8%	96.2%	
Quintile 3	2.8%	97.2%	
Quintile 4	2.5%	97.5%	
Quintile 5 - Least deprived	1.1%	98.9%	**<0.001**

Significant associations as determined by the chi-squared test of association are denoted in bold text

Supplementary table S3: Ordinal logistic regression analyses predicting food security status of differing severity, Block 1, showing odds ratios and standard errors, n=17,843

	**High vs marginal, low and very low food security**	**High and marginal vs low and very low food security**	**High, marginal and low vs very low food security**
Survey wave			
Wave 4 (Oct 2021-Jan 2022)	1.00 [1.00,1.00]	1.00 [1.00,1.00]	1.00 [1.00,1.00]
Wave 5 (April - July 2022)	1.20* [1.04,1.38]	1.20* [1.04,1.38]	1.20* [1.04,1.38]
Wave 6 (Oct 2022 - Jan 2023)	*1.51*** [1.31,1.75]*	*1.54*** [1.30,1.81]*	*1.61*** [1.30,2.00]*
Gender			
Male	1.00 [1.00,1.00]	1.00 [1.00,1.00]	1.00 [1.00,1.00]
Female	1.11 [1.00,1.23]	1.11 [1.00,1.23]	1.11 [1.00,1.23]
Age group			
16-24	1.00 [1.00,1.00]	1.00 [1.00,1.00]	1.00 [1.00,1.00]
25-34	0.77* [0.60,0.97]	0.77* [0.60,0.97]	0.77* [0.60,0.97]
35-44	0.55*** [0.44,0.70]	0.55*** [0.44,0.70]	0.55*** [0.44,0.70]
45-54	0.43*** [0.34,0.54]	0.43*** [0.34,0.54]	0.43*** [0.34,0.54]
55-64	0.30*** [0.24,0.38]	0.30*** [0.24,0.38]	0.30*** [0.24,0.38]
65-74	0.21*** [0.16,0.26]	0.21*** [0.16,0.26]	0.21*** [0.16,0.26]
75 +	*0.17*** [0.12,0.23]*	*0.12*** [0.08,0.18]*	*0.06*** [0.03,0.11]*
Household composition			
One adult no children	1.00 [1.00,1.00]	1.00 [1.00,1.00]	1.00 [1.00,1.00]
Couple no children	0.57*** [0.48,0.66]	0.57*** [0.48,0.66]	0.57*** [0.48,0.66]
Couple with children	0.78* [0.64,0.94]	0.78* [0.64,0.94]	0.78* [0.64,0.94]
Lone parent	2.53*** [1.88,3.41]	2.53*** [1.88,3.41]	2.53*** [1.88,3.41]

Other no children	0.56*** [0.46,0.69]	0.56*** [0.46,0.69]	0.56*** [0.46,0.69]
Other with children	0.93 [0.71,1.22]	0.93 [0.71,1.22]	0.93 [0.71,1.22]
Ethnicity			
White	1.00 [1.00,1.00]	1.00 [1.00,1.00]	1.00 [1.00,1.00]
Mixed	1.97*** [1.39,2.79]	1.97*** [1.39,2.79]	1.97*** [1.39,2.79]
Asian or Asian British	*1.49*** *[1.15,1.92]*	*1.36** *[1.02,1.80]*	*0.76* *[0.51,1.14]*
Black or Black British	1.31 [0.91,1.88]	1.31 [0.91,1.88]	1.31 [0.91,1.88]
Other ethnic group	1.30 [0.64,2.65]	1.30 [0.64,2.65]	1.30 [0.64,2.65]

* p < 0.05, ** p < 0.01, *** p < 0.001

Coefficients that do not satisfy the proportional odds assumption and that have therefore been allowed to vary across different levels of food security have been denoted by italics

Supplementary table S4: Ordinal logistic regression analyses predicting food security status of differing severity, Block 2 showing odds ratios and standard errors, n=17,843

	**High vs marginal, low and very low food security**	**High and marginal vs low and very low food security**	**High, marginal and low vs very low food security**
Survey wave			
Wave 4 (Oct 2021-Jan 2022)	1.00 [1.00,1.00]	1.00 [1.00,1.00]	1.00 [1.00,1.00]
Wave 5 (April - July 2022)	1.27** [1.10,1.48]	1.27** [1.10,1.48]	1.27** [1.10,1.48]
Wave 6 (Oct 2022 - Jan 2023)	*1.68*** [1.44,1.97]*	*1.67*** [1.40,1.99]*	*1.71*** [1.36,2.14]*
Gender			
Male	1.00 [1.00,1.00]	1.00 [1.00,1.00]	1.00 [1.00,1.00]
Female	0.96 [0.86,1.08]	0.96 [0.86,1.08]	0.96 [0.86,1.08]
Age group			
16-24	1.00 [1.00,1.00]	1.00 [1.00,1.00]	1.00 [1.00,1.00]
25-34	0.83 [0.61,1.13]	0.83 [0.61,1.13]	0.83 [0.61,1.13]
35-44	0.67** [0.49,0.90]	0.67** [0.49,0.90]	0.67** [0.49,0.90]
45-54	0.46*** [0.34,0.62]	0.46*** [0.34,0.62]	0.46*** [0.34,0.62]
55-64	0.25*** [0.18,0.34]	0.25*** [0.18,0.34]	0.25*** [0.18,0.34]
65-74	0.24*** [0.17,0.33]	0.24*** [0.17,0.33]	0.24*** [0.17,0.33]
75 +	*0.21*** [0.15,0.32]*	*0.16*** [0.10,0.25]*	*0.09*** [0.04,0.18]*
Household composition			
One adult no children	1.00 [1.00,1.00]	1.00 [1.00,1.00]	1.00 [1.00,1.00]
Couple no children	0.91 [0.78,1.06]	0.91 [0.78,1.06]	0.91 [0.78,1.06]
Couple with children	1.30** [1.07,1.59]	1.30** [1.07,1.59]	1.30** [1.07,1.59]
Lone parent	2.31*** [1.71,3.11]	2.31*** [1.71,3.11]	2.31*** [1.71,3.11]

Other no children	0.99 [0.80,1.24]	0.99 [0.80,1.24]	0.99 [0.80,1.24]
Other with children	1.63*** [1.23,2.16]	1.63*** [1.23,2.16]	1.63*** [1.23,2.16]
Ethnicity			
White	1.00 [1.00,1.00]	1.00 [1.00,1.00]	1.00 [1.00,1.00]
Mixed	1.90** [1.22,2.96]	1.90** [1.22,2.96]	1.90** [1.22,2.96]
Asian or Asian British	*1.08 [0.80,1.46]*	*0.98 [0.72,1.35]*	*0.53** [0.35,0.81]*
Black or Black British	1.19 [0.76,1.88]	1.19 [0.76,1.88]	1.19 [0.76,1.88]
Other ethnic group	0.94 [0.50,1.79]	0.94 [0.50,1.79]	0.94 [0.50,1.79]
Annual household income (non equivalised)			
Less than £19,000	1.00 [1.00,1.00]	1.00 [1.00,1.00]	1.00 [1.00,1.00]
£19,000 - £31,999	*0.40*** [0.34,0.47]*	*0.35*** [0.30,0.42]*	*0.39*** [0.31,0.49]*
£32,000 - £63,999	*0.16*** [0.14,0.19]*	*0.13*** [0.11,0.16]*	*0.13*** [0.10,0.17]*
£64,000 and above	*0.05*** [0.04,0.07]*	*0.03*** [0.02,0.05]*	*0.01*** [0.01,0.03]*
Employment status			
Working	1.00 [1.00,1.00]	1.00 [1.00,1.00]	1.00 [1.00,1.00]
Student	0.46*** [0.31,0.67]	0.46*** [0.31,0.67]	0.46*** [0.31,0.67]
Retired	0.38*** [0.32,0.47]	0.38*** [0.32,0.47]	0.38*** [0.32,0.47]
Unemployed	1.79** [1.20,2.68]	1.79** [1.20,2.68]	1.79** [1.20,2.68]
Unable to work due to poor health	2.13*** [1.58,2.88]	2.13*** [1.58,2.88]	2.13*** [1.58,2.88]
Homemaker	1.11 [0.88,1.40]	1.11 [0.88,1.40]	1.11 [0.88,1.40]
Other	1.02 [0.76,1.36]	1.02 [0.76,1.36]	1.02 [0.76,1.36]

* p < 0.05, ** p < 0.01, *** p < 0.001

Coefficients that do not satisfy the proportional odds assumption and that have therefore been allowed to vary across different levels of food security have been denoted by italics

Supplementary table S5: Ordinal logistic regression analyses predicting food security status of differing severity, Block 3 showing odds ratios and standard errors, n=17,843

	**High vs marginal, low and very low food security**	**High and marginal vs low and very low food security**	**High, marginal and low vs very low food security**
Survey wave			
Wave 4 (Oct 2021-Jan 2022)	1.00 [1.00,1.00]	1.00 [1.00,1.00]	1.00 [1.00,1.00]
Wave 5 (April - July 2022)	1.31*** [1.13,1.52]	1.31*** [1.13,1.52]	1.31*** [1.13,1.52]
Wave 6 (Oct 2022 - Jan 2023)	*1.74*** [1.49,2.03]*	*1.73*** [1.46,2.06]*	*1.82*** [1.46,2.28]*
Gender			
Male	1.00 [1.00,1.00]	1.00 [1.00,1.00]	1.00 [1.00,1.00]
Female	0.92 [0.82,1.03]	0.92 [0.82,1.03]	0.92 [0.82,1.03]
Age group			
16-24	1.00 [1.00,1.00]	1.00 [1.00,1.00]	1.00 [1.00,1.00]
25-34	0.81 [0.60,1.11]	0.81 [0.60,1.11]	0.81 [0.60,1.11]
35-44	0.66** [0.49,0.90]	0.66** [0.49,0.90]	0.66** [0.49,0.90]
45-54	0.45*** [0.33,0.61]	0.45*** [0.33,0.61]	0.45*** [0.33,0.61]
55-64	0.24*** [0.18,0.33]	0.24*** [0.18,0.33]	0.24*** [0.18,0.33]
65-74	0.22*** [0.16,0.31]	0.22*** [0.16,0.31]	0.22*** [0.16,0.31]
75 +	*0.19*** [0.13,0.28]*	*0.14*** [0.09,0.22]*	*0.08*** [0.04,0.16]*
Household composition			
One adult no children	1.00 [1.00,1.00]	1.00 [1.00,1.00]	1.00 [1.00,1.00]
Couple no children	0.93 [0.80,1.09]	0.93 [0.80,1.09]	0.93 [0.80,1.09]
Couple with children	1.43*** [1.17,1.75]	1.43*** [1.17,1.75]	1.43*** [1.17,1.75]
Lone parent	2.40*** [1.75,3.30]	2.40*** [1.75,3.30]	2.40*** [1.75,3.30]

Other no children	1.01 [0.81,1.26]	1.01 [0.81,1.26]	1.01 [0.81,1.26]
Other with children	1.73*** [1.31,2.30]	1.73*** [1.31,2.30]	1.73*** [1.31,2.30]
Ethnicity			
White	1.00 [1.00,1.00]	1.00 [1.00,1.00]	1.00 [1.00,1.00]
Mixed	1.80** [1.17,2.77]	1.80** [1.17,2.77]	1.80** [1.17,2.77]
Asian or Asian British	*1.15 [0.85,1.55]*	*1.10 [0.81,1.51]*	*0.62* [0.41,0.94]*
Black or Black British	1.36 [0.86,2.17]	1.36 [0.86,2.17]	1.36 [0.86,2.17]
Other ethnic group	0.92 [0.46,1.84]	0.92 [0.46,1.84]	0.92 [0.46,1.84]
Annual household income (non equivalised)			
Less than £19,000	1.00 [1.00,1.00]	1.00 [1.00,1.00]	1.00 [1.00,1.00]
£19,000 - £31,999	*0.40*** [0.34,0.47]*	*0.36*** [0.30,0.43]*	*0.41*** [0.33,0.52]*
£32,000 - £63,999	*0.16*** [0.14,0.19]*	*0.14*** [0.11,0.17]*	*0.14*** [0.10,0.18]*
£64,000 and above	*0.05*** [0.04,0.06]*	*0.04*** [0.02,0.05]*	*0.01*** [0.01,0.03]*
Employment status			
Working	1.00 [1.00,1.00]	1.00 [1.00,1.00]	1.00 [1.00,1.00]
Student	0.43*** [0.30,0.63]	0.43*** [0.30,0.63]	0.43*** [0.30,0.63]
Retired	0.36*** [0.30,0.44]	0.36*** [0.30,0.44]	0.36*** [0.30,0.44]
Unemployed	1.51* [1.03,2.21]	1.51* [1.03,2.21]	1.51* [1.03,2.21]
Unable to work due to poor health	1.30 [0.96,1.77]	1.30 [0.96,1.77]	1.30 [0.96,1.77]
Homemaker	1.01 [0.81,1.27]	1.01 [0.81,1.27]	1.01 [0.81,1.27]
Other	1.01 [0.74,1.38]	1.01 [0.74,1.38]	1.01 [0.74,1.38]
Long-term health condition status			

No long-term health condition	1.00 [1.00,1.00]	1.00 [1.00,1.00]	1.00 [1.00,1.00]
Has a long-term health condition	*1.74*** [1.52,1.99]*	*2.12*** [1.82,2.48]*	*2.51*** [2.03,3.09]*
Food hypersensitivity status			
No food hypersensitivity	1.00 [1.00,1.00]	1.00 [1.00,1.00]	1.00 [1.00,1.00]
Has a food hypersensitivity	1.19* [1.01,1.40]	1.17 [0.98,1.40]	1.49** [1.17,1.90]

* p < 0.05, ** p < 0.01, *** p < 0.001

Coefficients that do not satisfy the proportional odds assumption and that have therefore been allowed to vary across different levels of food security have been denoted by italics

Supplementary table S6: Logistic regression analyses predicting food security status from interactions with country, showing odds ratios and standard errors, n=13,271

	**Country and survey wave**	**Country and gender**	**Country and age group**	**Country and presence of children**	**Country and ethnicity**	**Country and income**	**Country and work status**	**Country and LTHCs**	**Country and hypersensitivity**	**Country and urban-rural status**	**Country and deprivation**
Intercept	1.19 [0.83,1.71]	1.17 [0.82,1.68]	1.18 [0.82,1.69]	1.17 [0.82,1.68]	1.17 [0.82,1.68]	1.18 [0.82,1.69]	1.18 [0.82,1.70]	1.17 [0.81,1.67]	1.17 [0.82,1.68]	1.17 [0.82,1.68]	1.19 [0.83,1.72]
Main effects											
England	1.00 [.,.]	1.00 [.,.]	1.00 [.,.]	1.00 [.,.]	1.00 [.,.]	1.00 [.,.]	1.00 [.,.]	1.00 [.,.]	1.00 [.,.]	1.00 [.,.]	1.00 [.,.]
Wales	0.98 [0.72,1.35]	1.24 [0.95,1.62]	1.25 [0.88,1.76]	1.23 [0.98,1.54]	1.25* [1.04,1.49]	1.19 [0.90,1.57]	1.08 [0.87,1.34]	1.34* [1.06,1.70]	1.26* [1.04,1.53]	1.36** [1.10,1.68]	1.09 [0.74,1.62]
Northern Ireland	0.92 [0.69,1.24]	0.94 [0.73,1.22]	0.86 [0.63,1.19]	1.02 [0.82,1.26]	0.99 [0.83,1.18]	0.93 [0.72,1.21]	0.92 [0.75,1.13]	0.97 [0.78,1.20]	0.96 [0.79,1.16]	0.85 [0.69,1.05]	0.68* [0.47,0.98]
Interaction terms											
Country and survey wave											
Wales x wave 4	1.00 [.,.]										
Wales x wave 5	1.63* [1.07,2.50]										
Wales x wave 6	1.11 [0.72,1.71]										

NI x wave 4	1.00 [.,.]										
NI x wave 5	1.11 [0.74,1.65]										
NI x wave 6	1.03 [0.68,1.55]										
Country and gender											
Wales x male		1.00 [.,.]									
Wales x female		0.98 [0.71,1.36]									
NI x male		1.00 [.,.]									
NI x female		1.05 [0.77,1.43]									
Country and age group											
Wales x 26-34 years			1.00 [.,.]								
Wales x 35-54 years			0.96 [0.63,1.45]								
Wales x 55-74 years			1.00 [0.64,1.54]								

Wales x 75+ years			1.08 [0.47,2.45]								
NI x 26-34 years			1.00 [.,.]								
NI x 35-54 years			1.16 [0.79,1.71]								
NI x 55-74 years			1.29 [0.85,1.95]								
NI x 75+ years			0.91 [0.37,2.24]								
Country and presence of children											
Wales x no children				1.00 [.,.]							
Wales x has children				0.99 [0.70,1.41]							
NI x no children				1.00 [.,.]							
NI x has children				0.87 [0.62,1.22]							
Country and ethnicity											
Wales x White					1.00 [.,.]						

Wales x ethnic minority					0.77 [0.32,1.86]						
NI x White					1.00 [.,.]						
NI x ethnic minority					0.37* [0.15,0.93]						
Country and income											
Wales x <£19,000						1.00 [.,.]					
Wales x £19,000 - £31,999						1.22 [0.81,1.83]					
Wales x £32,000 +						0.92 [0.60,1.40]					
NI x <£19,000						1.00 [.,.]					
NI x £19,000 - £31,999						0.96 [0.65,1.41]					
NI x £32,000 +						1.18 [0.81,1.72]					
Country and working status											

Wales x Working							1.00 [.,.]				
Wales x Student							1.45 [0.59,3.55]				
Wales x Retired							1.44 [0.96,2.15]				
Wales x Unemployed							1.17 [0.45,3.02]				
Wales x Unable to work due to poor health							1.80 [0.95,3.40]				
Wales x Homemaker							0.93 [0.50,1.74]				
Wales x Other							1.66 [0.72,3.84]				
NI x Working							1.00 [.,.]				
NI x Student							1.16 [0.55,2.45]				
NI x Retired							1.36 [0.90,2.05]				

NI x Unemployed							0.65 [0.27,1.59]				
NI x Unable to work due to poor health							1.29 [0.74,2.26]				
NI x Homemaker							1.12 [0.59,2.11]				
NI x Other							1.27 [0.56,2.88]				
Country and long-term health condition (LTHC)											
Wales x No LTHC								1.00 [.,.]			
Wales x Has LTHC								0.79 [0.56,1.11]			
NI x No LTHC								1.00 [.,.]			
NI x Has LTHC								1.00 [0.72,1.40]			
Country and food hypersensitivity											
Wales x No hyper.									1.00 [.,.]		

Wales x Has hyper.									0.83 [0.54,1.29]		
NI x No hyper.									1.00 [.,.]		
NI x Has hyper.									1.04 [0.70,1.54]		
Country and urban-rural status											
Wales x Urban area										1.00 [.,.]	
Wales x Rural area										0.69* [0.47,1.00]	
NI x Urban area										1.00 [.,.]	
NI x Rural area										1.34 [0.94,1.91]	
Country and deprivation											
Wales x Decile 1											1.00 [.,.]
Wales x Decile 2											1.60 [0.94,2.71]
Wales x Decile 3											1.13 [0.66,1.93]

Wales x Decile 4											0.97 [0.55,1.71]
Wales x Decile 5											0.95 [0.54,1.69]
NI x Decile 1											1.00 [.,.]
NI x Decile 2											2.18** [1.33,3.58]
NI x Decile 3											1.17 [0.70,1.97]
NI x Decile 4											1.78* [1.07,2.99]
NI x Decile 5											1.46 [0.87,2.45]

All models are also adjusted for main effects of covariates, not listed for brevity

Supplementary table S7: Logistic regression analyses predicting emergency food receipt from interactions with country, showing odds ratios and standard errors, n=8,730

	**Country and survey wave**	**Country and gender**	**Country and FI status**	**Country and age group**	**Country and presence of children**	**Country and ethnicity**	**Country and income**	**Country and work status**	**Country and LTHCs**	**Country and hypersensitivity**	**Country and urban-rural status**	**Country and deprivation**
Intercept	0.09*** [0.03,0.24]	0.09*** [0.03,0.23]	0.09*** [0.03,0.25]	0.09*** [0.03, 0.24]	0.09*** [0.03,0.25]	0.09*** [0.03,0.23]	0.09*** [0.03,0.24]	0.08*** [0.03,0.23]	0.09*** [0.03,0.24]	0.09*** [0.03,0.23]	0.09*** [0.03,0.24]	0.09*** [0.03,0.24]
Main effects												
England	1.000 [.,.]	1.000 [.,.]	1.000 [.,.]	1.000 [.,.]	1.000 [.,.]	1.000 [.,.]	1.000 [.,.]	1.000 [.,.]	1.000 [.,.]	1.000 [.,.]	1.000 [.,.]	1.000 [.,.]
Wales	0.50 [0.23,1.10]	0.97 [0.46,2.04]	0.36 [0.11,1.24]	0.77 [0.33, 1.81]	0.89 [0.49,1.60]	0.89 [0.54,1.47]	0.72 [0.41,1.29]	1.34 [0.74,2.43]	0.83 [0.37,1.86]	0.81 [0.48,1.39]	0.79 [0.45,1.39]	0.46 [0.21,1.02]
Northern Ireland	0.98 [0.43,2.24]	1.52 [0.77,3.00]	0.59 [0.26,1.38]	1.06 [0.51, 2.21]	1.53 [0.85,2.74]	1.26 [0.79,2.03]	0.93 [0.53,1.64]	1.60 [0.89,2.88]	1.07 [0.54,2.12]	1.18 [0.72,1.93]	1.09 [0.62,1.93]	0.66 [0.30,1.46]
Interaction terms												
Country and survey wave												
Wales x wave 4	1.00 [.,.]											
Wales x wave 5	2.62 [0.80,8.58]											
Wales x wave 6	1.95 [0.69,5.52]											

NI x wave 4	1.00 [.,.]											
NI x wave 5	1.97 [0.70,5.54]											
NI x wave 6	1.02 [0.35,2.99]											
Country and gender												
Wales x male		1.00 [.,.]										
Wales x female		0.80 [0.33,1.92]										
NI x male		1.00 [.,.]										
NI x female		0.69 [0.31,1.51]										
Country and food security status												
Wales x food secure			1.00 [.,.]									
Wales x food insecure			2.91 [0.78,10.93]									

NI x food secure			1.00 [.,.]									
NI x food insecure			2.65* [1.02,6.93]									
Country and age group												
Wales x 26-34 years				1.00 [.,.]								
Wales x 35-54 years				1.61 [0.61, 4.23]								
Wales x 55-74 years				0.41 [0.09, 1.84]								
Wales x 75+ years				1.00 [.,.]								
NI x 26-34 years				1.00 [.,.]								
NI x 35-54 years				1.04 [0.41, 2.64]								
NI x 55-74 years				1.89 [0.64, 5.57]								

NI x 75+ years				0.60 [0.05, 8.00]								
Country and presence of children												
Wales x no children					1.00 [.,.]							
Wales x has children					0.91 [0.35,2.40]							
NI x no children					1.00 [.,.]							
NI x has children					0.61 [0.27,1.38]							
Country and ethnicity												
Wales x White						1.00 [.,.]						
Wales x ethnic minority						0.64 [0.11,3.76]						
NI x White						1.00 [.,.]						
NI x ethnic						0.51 [0.08,3.35]						

minority												
Country and income												
Wales x <£19,000							1.00 [.,.]					
Wales x £19,000-£31,999							1.76 [0.61,5.10]					
Wales x £32,000+							2.42 [0.53,10.92]					
NI x <£19,000							1.00 [.,.]					
NI x £19,000-£31,999							1.91 [0.81,4.47]					
NI x £32,000+							4.57* [1.22,17.06]					
Country and working status												
Wales x Working								1.00 [.,.]				

Wales x Student								1.01 [0.23,4.47]				
Wales x Retired								0.15* [0.03,0.89]				
Wales x Unemployed								0.17* [0.04,0.74]				
Wales x Sick								0.59 [0.20,1.72]				
Wales x Home maker								0.40 [0.11,1.48]				
Wales x Other								0.54 [0.09,3.33]				
NI x Working								1.00 [.,.]				
NI x Student								0.49 [0.11,2.25]				
NI x Retired								1.82 [0.52,6.42]				
NI x Unemployed								0.33 [0.09,1.22]				

NI x Sick								0.29 [0.08,1.13]				
NI x Home maker								0.94 [0.27,3.27]				
NI x Other								1.00 [.,.]				
Country and long-term health condition (LTHC)												
Wales x No LTHC									1.00 [.,.]			
Wales x Has LTHC									1.05 [0.38,2.93]			
NI x No LTHC									1.00 [.,.]			
NI x Has LTHC									1.36 [0.55,3.36]			
Country and food hypersensitivity												
Wales x No hyper.										1.00 [.,.]		
Wales x Has hyper.										1.32 [0.41,4.22]		
NI x No hyper.										1.00 [.,.]		

NI x Has hyper.										1.27 [0.46,3.48]		
Country and urban-rural status												
Wales x Urban area											1.00 [.,.]	
Wales x Rural area											1.41 [0.46,4.28]	
NI x Urban area											1.00 [.,.]	
NI x Rural area											1.41 [0.53,3.76]	
Country and deprivation												
Wales x Decile 1												1.00 [.,.]
Wales x Decile 2												5.78** [1.92,17.38]
Wales x Decile 3												3.05 [0.84,11.09]
Wales x Decile 4												1.73 [0.33,9.18]

Wales x Decile 5												0.95 [0.18,4.98]
NI x Decile 1												1.00 [.,.]
NI x Decile 2												5.05** [1.67,15.31]
NI x Decile 3												1.19 [0.32,4.44]
NI x Decile 4												3.23 [0.89,11.78]
NI x Decile 5												0.90 [0.21,3.82]

All models are also adjusted for main effects of covariates, not listed for brevity

Supplementary table S8: Logistic regression analyses predicting emergency food receipt from very low food security and other predictors, showing odds ratios and standard errors, n=11,161

	**Bivariate associations**	**Block 1: Demographics**	**Block 2: Demographics plus financial characteristics**	**Block 3: Demographics plus financial characteristics plus health**	**Block 4: Demographics plus financial characteristics plus health plus local characteristics**
Intercept	Variable, from 0.01 to 0.12	0.07*** [0.03,0.16]	0.11*** [0.04,0.27]	0.10*** [0.04,0.26]	0.14*** [0.05,0.37]
Survey wave					
Wave 4 (Oct 2021-Jan 2022)	1.00 [.,.]	1.00 [.,.]	1.00 [.,.]	1.00 [.,.]	1.00 [.,.]
Wave 5 (April - July 2022)	0.72 [0.46,1.12]	0.59* [0.36,0.95]	0.62 [0.38,1.03]	0.62 [0.37,1.04]	0.62 [0.38,1.02]
Wave 6 (Oct 2022 - Jan 2023)	0.85 [0.53,1.38]	0.58* [0.34,0.97]	0.65 [0.38,1.11]	0.66 [0.38,1.13]	0.68 [0.40,1.15]
Food security status					
High, marginal or low food security	1.00 [.,.]	1.00 [.,.]	1.00 [.,.]	1.00 [.,.]	1.00 [.,.]
Very low food security	15.02*** [10.16,22.21]	13.45*** [9.13,19.80]	5.56*** [3.59,8.61]	5.12*** [3.33,7.87]	5.20*** [3.40,7.94]
Gender					
Male	1.00 [.,.]	1.00 [.,.]	1.00 [.,.]	1.00 [.,.]	1.00 [.,.]
Female	1.08 [0.76,1.54]	1.06 [0.73,1.55]	0.94 [0.63,1.40]	0.92 [0.61,1.38]	0.94 [0.63,1.41]
Age group					
16-24	1.00 [.,.]	1.00 [.,.]	1.00 [.,.]	1.00 [.,.]	1.00 [.,.]
25-34	0.42** [0.24,0.76]	0.37** [0.19,0.71]	0.38* [0.18,0.81]	0.38* [0.18,0.81]	0.36** [0.17,0.76]
35-44	0.51* [0.30,0.89]	0.53* [0.28,0.99]	0.58 [0.28,1.17]	0.57 [0.28,1.19]	0.57 [0.28,1.16]
45-54	0.33*** [0.19,0.59]	0.44** [0.24,0.82]	0.46* [0.21,1.00]	0.46 [0.21,1.01]	0.48 [0.22,1.05]

55-64	0.22***<br>[0.11,0.42]	0.36**<br>[0.18,0.73]	0.33**<br>[0.15,0.74]	0.32**<br>[0.14,0.73]	0.36*<br>[0.16,0.82]
65-74	0.10***<br>[0.04,0.23]	0.22**<br>[0.09,0.55]	0.37<br>[0.10,1.37]	0.36<br>[0.10,1.34]	0.38<br>[0.10,1.42]
75 +	0.20*<br>[0.05,0.75]	0.39<br>[0.09,1.72]	0.84<br>[0.15,4.51]	0.75<br>[0.13,4.21]	0.87<br>[0.16,4.78]
Household composition					
One adult no children	1.00<br>[.,.]	1.00<br>[.,.]	1.00<br>[.,.]	1.00<br>[.,.]	1.00<br>[.,.]
Couple no children	0.54*<br>[0.32,0.89]	0.62<br>[0.35,1.10]	0.91<br>[0.52,1.60]	0.92<br>[0.52,1.62]	0.91<br>[0.52,1.58]
Couple with children	1.09<br>[0.65,1.86]	0.72<br>[0.37,1.40]	1.23<br>[0.62,2.43]	1.27<br>[0.64,2.54]	1.36<br>[0.68,2.71]
Lone parent	4.20***<br>[2.26,7.80]	1.60<br>[0.72,3.58]	1.77<br>[0.75,4.16]	1.81<br>[0.76,4.31]	1.94<br>[0.82,4.57]
Other no children	0.84<br>[0.43,1.67]	0.54<br>[0.26,1.14]	0.91<br>[0.43,1.90]	0.90<br>[0.43,1.90]	0.94<br>[0.47,1.89]
Other with children	1.09<br>[0.51,2.30]	0.43<br>[0.18,1.05]	0.72<br>[0.31,1.70]	0.71<br>[0.30,1.68]	0.72<br>[0.30,1.71]
Ethnicity					
White	1.00<br>[.,.]	1.00<br>[.,.]	1.00<br>[.,.]	1.00<br>[.,.]	1.00<br>[.,.]
Ethnic minority	2.39**<br>[1.41,4.05]	2.42**<br>[1.34,4.37]	1.87*<br>[1.03,3.40]	1.95*<br>[1.07,3.55]	1.83<br>[0.97,3.46]
Household income					
Less than £19,000	1.00<br>[.,.]		1.00<br>[.,.]	1.00<br>[.,.]	1.00<br>[.,.]
£19,000 - £31,999	0.22***<br>[0.13,0.37]		0.40**<br>[0.22,0.72]	0.40**<br>[0.22,0.72]	0.42**<br>[0.23,0.76]
£32,000 and above	0.05***<br>[0.03,0.12]		0.14***<br>[0.05,0.36]	0.14***<br>[0.05,0.36]	0.16***<br>[0.06,0.42]
Employment status					
Working	1.00<br>[.,.]		1.00<br>[.,.]	1.00<br>[.,.]	1.00<br>[.,.]

Student	3.89***<br>[1.90,7.96]		1.03<br>[0.38,2.78]	1.00<br>[0.36,2.79]	1.09<br>[0.40,3.00]
Retired	0.47<br>[0.21,1.05]		0.47<br>[0.16,1.37]	0.45<br>[0.15,1.34]	0.49<br>[0.16,1.50]
Unemployed	12.86***<br>[7.38,22.38]		3.16***<br>[1.69,5.92]	2.91**<br>[1.52,5.54]	3.10***<br>[1.61,5.95]
Unable to work due to poor health	13.77***<br>[8.54,22.22]		4.04***<br>[2.31,7.06]	3.21***<br>[1.67,6.18]	3.26***<br>[1.67,6.36]
Homemaker	4.48***<br>[2.59,7.74]		1.94<br>[0.92,4.11]	1.82<br>[0.85,3.91]	1.87<br>[0.86,4.06]
Other	4.47***<br>[2.28,8.80]		2.53*<br>[1.20,5.31]	2.49*<br>[1.19,5.22]	2.44*<br>[1.10,5.40]
Long-term health condition status					
No long-term health condition	1.00<br>[.,.]			1.00<br>[.,.]	1.00<br>[.,.]
Has a long-term health condition	2.92***<br>[1.98,4.31]			1.47<br>[0.89,2.44]	1.40<br>[0.84,2.32]
Food hypersensitivity status					
No food hypersensitivity	1.00<br>[.,.]			1.00<br>[.,.]	1.00<br>[.,.]
Has a food hypersensitivity	1.18<br>[0.74,1.88]			0.95<br>[0.54,1.68]	0.97<br>[0.56,1.68]
Urban/rural classification					
Urban	1.00<br>[.,.]				1.00<br>[.,.]
Rural	0.55*<br>[0.32,0.95]				1.15<br>[0.65,2.05]
Country					
England	1.00<br>[.,.]				1.00<br>[.,.]
Wales	1.06<br>[0.74,1.50]				0.91<br>[0.60,1.37]
Northern Ireland	1.27<br>[0.92,1.76]				1.43<br>[0.96,2.14]
Index of Multiple Deprivation (IMD)					

Quintile 1 (most deprived)	1.00<br>[.,.]				1.00<br>[.,.]
Quintile 2	0.37***<br>[0.24,0.58]				0.43**<br>[0.26,0.72]
Quintile 3	0.34***<br>[0.19,0.60]				0.55<br>[0.28,1.07]
Quintile 4	0.22***<br>[0.10,0.48]				0.52<br>[0.22,1.21]
Quintile 5 (least deprived)	0.11***<br>[0.05,0.24]				0.33**<br>[0.14,0.75]

* p < 0.05, ** p < 0.01, *** p < 0.001

Supplementary table S9: Logistic regression analyses predicting emergency food receipt, without controlling for food security status, showing odds ratios and standard errors, n=11,161

	**Bivariate associations**	**Block 1: Demographics**	**Block 2: Demographics plus financial characteristics**	**Block 3: Demographics plus financial characteristics plus health**	**Block 4: Demographics plus financial characteristics plus health plus local characteristics**
Intercept	Variable, from 0.01 to 0.12	0.19*** [0.09,0.39]	0.22** [0.08,0.59]	0.17*** [0.06,0.47]	0.24** [0.08,0.69]
Survey wave					
Wave 4 (Oct 2021-Jan 2022)	1.00 [.,.]	1.00 [.,.]	1.00 [.,.]	1.00 [.,.]	1.00 [.,.]
Wave 5 (April - July 2022)	0.72 [0.46,1.12]	0.71 [0.45,1.12]	0.74 [0.46,1.19]	0.73 [0.45,1.20]	0.75 [0.47,1.21]
Wave 6 (Oct 2022 - Jan 2023)	0.85 [0.53,1.38]	0.80 [0.50,1.29]	0.78 [0.46,1.31]	0.79 [0.46,1.33]	0.82 [0.49,1.38]
Gender					
Male	1.00 [.,.]	1.00 [.,.]	1.00 [.,.]	1.00 [.,.]	1.00 [.,.]
Female	1.08 [0.76,1.54]	1.09 [0.76,1.55]	0.90 [0.61,1.33]	0.86 [0.57,1.30]	0.87 [0.59,1.31]
Age group					
16-24	1.00 [.,.]	1.00 [.,.]	1.00 [.,.]	1.00 [.,.]	1.00 [.,.]
25-34	0.42** [0.24,0.76]	0.37** [0.20,0.68]	0.39* [0.18,0.84]	0.38* [0.17,0.83]	0.36* [0.16,0.79]
35-44	0.51* [0.30,0.89]	0.41** [0.23,0.73]	0.53 [0.25,1.11]	0.52 [0.24,1.12]	0.52 [0.24,1.11]
45-54	0.33*** [0.19,0.59]	0.31*** [0.18,0.54]	0.38* [0.17,0.83]	0.39* [0.17,0.87]	0.41* [0.18,0.91]
55-64	0.22*** [0.11,0.42]	0.23*** [0.12,0.42]	0.24*** [0.10,0.54]	0.23*** [0.10,0.54]	0.26** [0.11,0.60]
65-74	0.10*** [0.04,0.23]	0.10*** [0.04,0.23]	0.21* [0.06,0.74]	0.20* [0.06,0.74]	0.22* [0.06,0.80]
75 +	0.20* [0.05,0.75]	0.18* [0.05,0.72]	0.52 [0.10,2.64]	0.44 [0.08,2.40]	0.51 [0.10,2.71]
Household composition					

One adult no children	1.00 [.,.]	1.00 [.,.]	1.00 [.,.]	1.00 [.,.]	1.00 [.,.]
Couple no children	0.54* [0.32,0.89]	0.49** [0.29,0.83]	0.95 [0.54,1.65]	0.95 [0.54,1.66]	0.95 [0.54,1.65]
Couple with children	1.09 [0.65,1.86]	0.64 [0.34,1.21]	1.27 [0.66,2.44]	1.40 [0.73,2.71]	1.50 [0.78,2.90]
Lone parent	4.20*** [2.26,7.80]	2.57** [1.31,5.08]	2.50* [1.18,5.28]	2.51* [1.16,5.44]	2.66* [1.23,5.75]
Other no children	0.84 [0.43,1.67]	0.43* [0.21,0.87]	0.89 [0.43,1.83]	0.89 [0.43,1.83]	0.93 [0.46,1.87]
Other with children	1.09 [0.51,2.30]	0.43* [0.19,0.95]	0.89 [0.40,1.97]	0.87 [0.39,1.97]	0.90 [0.39,2.05]
Ethnicity					
White	1.00 [.,.]	1.00 [.,.]	1.00 [.,.]	1.00 [.,.]	1.00 [.,.]
Ethnic minority	2.39** [1.41,4.05]	1.81* [1.06,3.06]	1.43 [0.82,2.48]	1.58 [0.90,2.78]	1.47 [0.80,2.70]
Household income					
Less than £19,000	1.00 [.,.]		1.00 [.,.]	1.00 [.,.]	1.00 [.,.]
£19,000 - £31,999	0.22*** [0.13,0.37]		0.31*** [0.18,0.55]	0.33*** [0.19,0.57]	0.35*** [0.20,0.60]
£32,000 and above	0.05*** [0.03,0.12]		0.08*** [0.03,0.19]	0.08*** [0.03,0.21]	0.10*** [0.04,0.24]
Employment status					
Working	1.00 [.,.]		1.00 [.,.]	1.00 [.,.]	1.00 [.,.]
Student	3.89*** [1.90,7.96]		0.83 [0.30,2.26]	0.81 [0.29,2.31]	0.88 [0.31,2.49]
Retired	0.47 [0.21,1.05]		0.41 [0.16,1.10]	0.39 [0.14,1.08]	0.42 [0.15,1.19]
Unemployed	12.86*** [7.38,22.38]		4.45*** [2.39,8.28]	3.81*** [2.03,7.14]	4.11*** [2.20,7.68]
Unable to work due to poor health	13.77*** [8.54,22.22]		5.43*** [3.14,9.38]	3.47*** [1.84,6.52]	3.52*** [1.85,6.69]

Homemaker	4.48*** [2.59,7.74]		2.21* [1.11,4.40]	1.92 [0.94,3.92]	1.99 [0.97,4.06]
Other	4.47*** [2.28,8.80]		2.83** [1.37,5.85]	2.75** [1.34,5.67]	2.65* [1.22,5.75]
Long-term health condition status					
No long-term health condition	1.00 [.,.]			1.00 [.,.]	1.00 [.,.]
Has a long-term health condition	2.92*** [1.98,4.31]			2.05** [1.25,3.38]	1.92* [1.16,3.18]
Food hypersensitivity status					
No food hypersensitivity	1.00 [.,.]			1.00 [.,.]	1.00 [.,.]
Has a food hypersensitivity	1.18 [0.74,1.88]			1.03 [0.58,1.83]	1.06 [0.60,1.86]
Urban/rural classification					
Urban	1.00 [.,.]				1.00 [.,.]
Rural	0.55* [0.32,0.95]				1.16 [0.67,2.03]
Country					
England	1.00 [.,.]				1.00 [.,.]
Wales	1.06 [0.74,1.50]				0.97 [0.65,1.43]
Northern Ireland	1.27 [0.92,1.76]				1.31 [0.88,1.95]
Index of Multiple Deprivation (IMD)					
Quintile 1 (most deprived)	1.00 [.,.]				1.00 [.,.]
Quintile 2	0.37*** [0.24,0.58]				0.45** [0.27,0.75]
Quintile 3	0.34*** [0.19,0.60]				0.58 [0.31,1.08]
Quintile 4	0.22*** [0.10,0.48]				0.50 [0.22,1.14]
Quintile 5 (least deprived)	0.11*** [0.05,0.24]				0.33** [0.15,0.75]

$* p < 0.05$, $** p < 0.01$, $*** p < 0.001$
